# Progress in educational theory and translational implementation of simulation-based education for healthcare professionals: an integrative analysis from theory to effectiveness

**DOI:** 10.3389/fmed.2026.1947067

**Published:** 2026-09-10

**Authors:** Jiali Xu, Xiaoqin Yu, Zhen Fang, Xiaoying Wang, Liling Zhang, Yanting Cai

**Affiliations:** Affiliated Hospital of Jiujiang University, Jiujiang, Jiangxi, China

**Keywords:** competency-based medical education, debriefing, mastery learning, simulation-based education, translational simulation

## Abstract

**Aim:**

To synthesise pedagogical theory and contemporary evidence on simulation-based education (SBE) for healthcare professionals and translate this into practical design, implementation, and governance guidance.

**Design:**

Integrative narrative review.

**Methods:**

We performed dual-wave systematic literature retrieval across six academic databases (PubMed, CINAHL Complete, Web of Science Core Collection, Scopus, ERIC, Cochrane Library) from 1 January 2019 to 10 October 2025, supplemented by manual reference screening and expert consultation. Two independent reviewers completed title/abstract screening, full-text eligibility assessment, standardized 11-field data extraction and study quality appraisal using five validated risk-of-bias tools (MMAT 2018, SANRA, AGREE II, RoB 2, ROBINS-I). Extracted evidence was synthesized into a four-part analytic framework covering pedagogical foundations, instructional design, multi-level effectiveness evaluation and translational implementation governance. Salient constructs included experiential learning, deliberate practice, simulation-based mastery learning, cognitive load management, psychological safety, and objective-assessment alignment. We prioritized functional and psychological fidelity over high-end visual equipment, standardized structured briefing–simulation–debriefing cycles, and individualized mastery-based learning pacing in our analytical synthesis.

**Results:**

Across technical and non-technical domains, SBE improves knowledge, procedural skill, teamwork, and process outcomes; effect size and durability depend on design quality and debriefing. Prioritising functional/psychological fidelity over visual realism, aligning objectives with assessment, using SBML and spaced reinforcement, and embedding structured debriefing are consistent determinants of **effectiveness.** Digital and VR/AR modalities expand scale and analytics when cognitive load is managed. Translational simulation, guided by CFIR/RE-AIM and quality-improvement methods, links education to workflow reliability, latent safety threat detection, and early patient/process improvements. Evidence gaps persist for long-term retention, cost-effectiveness, and equity.

**Conclusions:**

SBE effectiveness is conditional on alignment of aims, activities, assessment, high-quality debriefings, mastery-based pacing, and psychologically safe climates. Functional and psychological fidelity should supersede device sophistication. Translational approaches position SBE within organisational quality and economic frameworks.

**Impact:**

This review offers a practical selection matrix and implementation governance model that assists educators in designing scalable and equitable SBE, supports administrators in resource planning and quality assurance, and guides accreditors in aligning evidence with entrustment and standards.

## 1 Introduction

Medical education is shifting from opportunistic clinical exposure toward structured capability development characterized by controllability, reproducibility, and measurable assessment. Driven by imperatives related to patient safety, competency-based education, and quality assurance, simulation-based education provides a low-risk, standardized, and traceable instructional cycle, applicable across both technical and non-technical skill domains and at individual and team levels (1). Recent studies and reviews indicate that technology-enhanced simulation methods—including high-fidelity mannequins, scenario-based simulations, and screen-based or virtual simulations—demonstrate broadly positive effects on learner knowledge acquisition, skills proficiency, and procedural efficiencies compared to traditional methods, with particularly pronounced effects on process-oriented outcomes such as operational success rates and efficiency (2). In team and communication skill development, interprofessional simulations moderately improve collaborative teamwork and communication competencies (3). At higher evaluation levels of the learning–behavior–outcome framework, pediatric emergency team simulation training has shown associations with improved adherence to time-critical actions and clinical guidelines, though evidence of direct impact on definitive patient outcomes such as survival remains suggestive and of limited certainty (4). Similarly, procedural skill simulations within mastery learning paradigms have provided preliminary evidence of associations between educational interventions and selected patient outcomes—for instance, a decreasing trend in infection rates in the context of left ventricular assist device management, though this finding derives from a single pilot study and should not be generalized (5). Consequently, the current discourse has evolved from questioning “whether simulation is effective” toward examining “under what conditions it is effective, how it should be designed and implemented, and what systemic outcomes it achieves.”.

In this review, simulation-based education is conceptualized as instructional practices that replicate clinical tasks and collaborative teamwork in controlled environments, thereby enabling standardized assessments and mastery learning outcomes. Fidelity is discussed as a multidimensional concept encompassing technical fidelity of simulation equipment, contextual fidelity relating to task sequence and environmental cues or distractions, and psychological fidelity reflecting learner immersion and engagement. Emphasis is placed on prioritizing functional and psychological fidelity rather than device sophistication (6). Crucially, simulation effectiveness hinges less upon equipment selection and more on aligning learning objectives, activities, and assessment strategies, alongside high-quality debriefings and psychological safety assurance. Recent systematic reviews advocate explicitly embedding elements such as psychological safety, role clarity, scenario boundaries, and evaluative feedback into structured debriefing frameworks, albeit noting that overall methodological rigor remains a key area for improvement (7, 8). Regarding digital formats, recent meta-analyses demonstrate positive skill-development outcomes, although lower-immersion interventions may sometimes be superior for knowledge acquisition, reinforcing the notion that instructional design and cognitive load management outweigh technological immersion alone (9). Furthermore, evidence from resource-constrained contexts highlights successful patient- and service-level improvements achievable with low-technology simulations emphasizing functional fidelity and scenario relevance, underscoring a pragmatic realism approach (10). While previous reviews have addressed individual components of SBE—pedagogical theory (11–13), instructional design (14, 15), modality-specific effectiveness (16–18), implementation frameworks (19, 20), or governance (21, 22)—no single prior review has integrated all four domains (theory, design, implementation, and governance/economics) into a coherent translational framework with operational tools. The present review makes three distinct contributions: (a) an integrated translational conceptual framework (Figure 2) that maps theory-design-implementation-effectiveness relationships across six domains; (b) an evidence-informed instructional selection matrix (Table 1) that operationally links clinical tasks, EPAs/MPSs, modalities, dose, assessment, and outcomes; and (c) a structured governance and economic evaluation model (Sections 6.4–6.5) that embeds simulation within institutional quality assurance and Learning Health System frameworks. A systematic comparison of the present review with 15 major prior reviews and frameworks is provided in Supplementary Table S1.

**Table 1:** Core alignment—individual/encounter-level simulation-based education.

Objective (EPA/MPS)	Modality & fidelity	Process & dose	Assessment	Target learner group	Follow-up period	Evidence type	Evidence quality	Outcomes/notes
CVC without complications; MPS: all critical steps;	Task trainer/arm; functional > visual; graded distractors	Pre-brief → drill → PEARLS; iterate to MPS; spaced boosters	OSATS; errors & time; IRR ≥ 0.8	Surgical & Internal Medicine Residents (PGY 1–3)	3-month skill retention follow-up	Randomized Controlled Crossover Trial; Systematic Review	High	Time/errors ↓; functional alignment outperforming superficial look (95); SBML effective (45)
Emergency cricothyrotomy; MPS; 0 critical errors	Procedure trainer; crisis micro-sims; functional cues	Rapid SBML; coached re-practice	Time; error tally; 1–3 mo retention	Emergency, Anesthesia, Surgical Residents; Advanced Practice Nurses	1–3 month skill retention follow-up	Randomized Controlled Trial; Systematic Review	High	Universal skill acquisition; large gains (27)
eFAST to standard; early proficiency	US task trainer/SP overlay; low → higher complexity	Scan-feedback cycles; MPS gate	Checklist; image-quality rating	Emergency Medicine, Radiology Residents; Clinical Clerkship Medical Students	2-month skill retention follow-up	Randomized Controlled Trial	High	RCT: SBML improves proficiency (26)
History-taking & SDM (AAMC EPA-1); empathy behaviors	SP with scripts; psychological fidelity	Pre-brief → encounter → structured debrief	Behavioral/communication ratings	Medical Students (pre-clinical & clinical clerkship years)	1-month post-training behavioral follow-up	Randomized Controlled Trial	Moderate	SP > peer role-play (50); consistent benefits (51); empathy ↑ (52)
IPC skills (hand hygiene, PPE); MPS; contamination = 0	Brief didactic + low-tech functional sim	Coached rehearsal; spaced refreshers	Direct observation; UV tracer	Medical Students, Residents, Inpatient Nurses		Systematic Review; Randomized Controlled Trial	High	SBL stronger for skills/attitudes; didactic suits knowledge (114)
Clinical reasoning (virtual patients); accuracy & time thresholds	Screen-based sim; ≥ 30 min; multiple variants; instant feedback	Attempt → feedback → re-attempt; spaced cases	Accuracy, time, sequence analytics	Medical Students, Residents across all clinical specialties	3-month clinical reasoning performance follow-up	Randomized Controlled Trial; Cohort Study	Moderate	Better reasoning with length/variants (78); analytics-enabled feedback (103, 111)
Chest tube insertion (low-cost); MPS; sterile technique	Low-cost reusable trainer; functional cues	Pre-brief → coached practice → MPS; retention checks	Steps/time; confidence	Emergency, Surgical Residents; Clinicians in resource-limited/LMIC settings	2-month skill retention follow-up	Quasi-experimental Study; Expert Consensus Guideline	Moderate	Realistic/effective low-cost model (64); supports equity (62, 63)

The following review questions guided this synthesis:

Primary review question: What are the relationships among pedagogical theory, instructional design variables, implementation strategies, and clinical/system-level outcomes in simulation-based education for healthcare professionals?.

Secondary review questions: (a) How do different simulation modalities, fidelity configurations, and instructional design features moderate educational and clinical outcomes across learner levels and clinical contexts? (b) What implementation science frameworks, economic evaluation approaches, and governance structures have been applied to simulation-based education, and how do they inform the translation of educational interventions into sustained clinical and organizational improvements? (c) What are the critical evidence gaps, methodological limitations, and priority research directions in the field of simulation-based healthcare education?

Ultimately, this review synthesizes fragmented evidence into a reusable analytical framework comprising explanation, operational guidance, and integration. This framework aims to support educators, administrators, and accreditation authorities in delivering evidence-based, scalable, and cost-effective simulation interventions, and aligns with emerging data analytics applications in educational debriefing and feedback processes (23, 24).

## 2 Methods

This review was conducted as an integrative review incorporating systematic search, screening, data extraction, and quality appraisal procedures. It is reported in alignment with the Preferred Reporting Items for Systematic Reviews and Meta-Analyses (PRISMA) 2020 statement, with adaptations appropriate for an integrative (rather than systematic-with-meta-analysis) review design. A completed PRISMA 2020 checklist is provided as Supplementary File S3.

### 2.1 Protocol registration

While no prospective publicly registered protocol existed at review initiation, a complete review protocol documenting pre-specified review questions, inclusion/exclusion criteria, search strategy, data extraction and quality-appraisal procedures has been compiled and is available within Supplementary Material. Deviations between *post-hoc* documented protocol and final synthesis are explicitly reported.

### 2.2 Search strategy

A systematic search was conducted across six electronic databases: PubMed, CINAHL (EBSCO), Scopus, Web of Science Core Collection, ERIC (ProQuest), and the Cochrane Library. The initial search was performed from January 2019 with a supplementary update on October 10, 2025. The Boolean search strategy combined controlled vocabulary (MeSH, CINAHL headings) and free-text terms across four conceptual blocks: (1) simulation-based education terms (e.g., “simulation-based education,” “simulation training,” “high-fidelity simulation,” “virtual simulation,” “standardized patient,” “task trainer,” “in situ simulation”); (2) healthcare professional terms (e.g., “healthcare professionals,” “medical education,” “nursing education,” “allied health,” “clinical education”); (3) pedagogical and design terms (e.g., “instructional design,” “experiential learning,” “deliberate practice,” “mastery learning,” “cognitive load,” “debriefing,” “feedback,” “assessment”); and (4) implementation and outcome terms (e.g., “implementation science,” “translational simulation,” “clinical transfer,” “patient outcomes,” “cost-effectiveness,” “governance,” “equity”). The complete search strategy for each database is provided in Supplementary File S1.

### 2.3 Inclusion and exclusion criteria

Inclusion criteria: (a) peer-reviewed quantitative, qualitative, or mixed-methods empirical studies; systematic, scoping, integrative, or narrative reviews; practice guidelines and professional standards; (b) addressing simulation-based education for healthcare professionals or health professions students at any training level; (c) reporting on at least one of the following: pedagogical theory application, instructional design, assessment methodology, implementation strategy, clinical transfer, patient outcomes, economic evaluation, or governance; (d) published in English between January 2019 and October 2025.

Exclusion criteria: (a) editorials, commentaries, opinion pieces, letters, or conference abstracts only; (b) studies focused exclusively on patient education or simulation used solely for patient treatment (e.g., surgical planning simulation without educational design elements); (c) studies reporting simulation use solely for high-stakes summative licensure or credentialing decisions without educational design elements; (d) studies for which full text was not available despite interlibrary loan requests. Note: Some references published after the formal database-search cutoff date (10 October 2025) are cited within this manuscript for comparative contextual discussion only. These post-search publications were manually identified during manuscript revision; they were not retrieved via database searching and did not undergo title-abstract/full-text screening, standardized data extraction, or risk-of-bias appraisal. They are excluded from the primary evidence corpus used for narrative evidence synthesis.

### 2.4 Study selection

Records were imported into Covidence systematic review software for deduplication and screening. Two reviewers independently screened titles and abstracts against the eligibility criteria. All records advanced by either reviewer proceeded to full-text review. The same two reviewers independently assessed full texts for eligibility. Disagreements were resolved through consensus discussion, with a third reviewer serving as arbiter when consensus could not be reached. Inter-rater agreement was calculated using Cohen's kappa (title/abstract screening: *κ* = 0.78; full-text screening: *κ* = 0.84).

**Figure 1:**
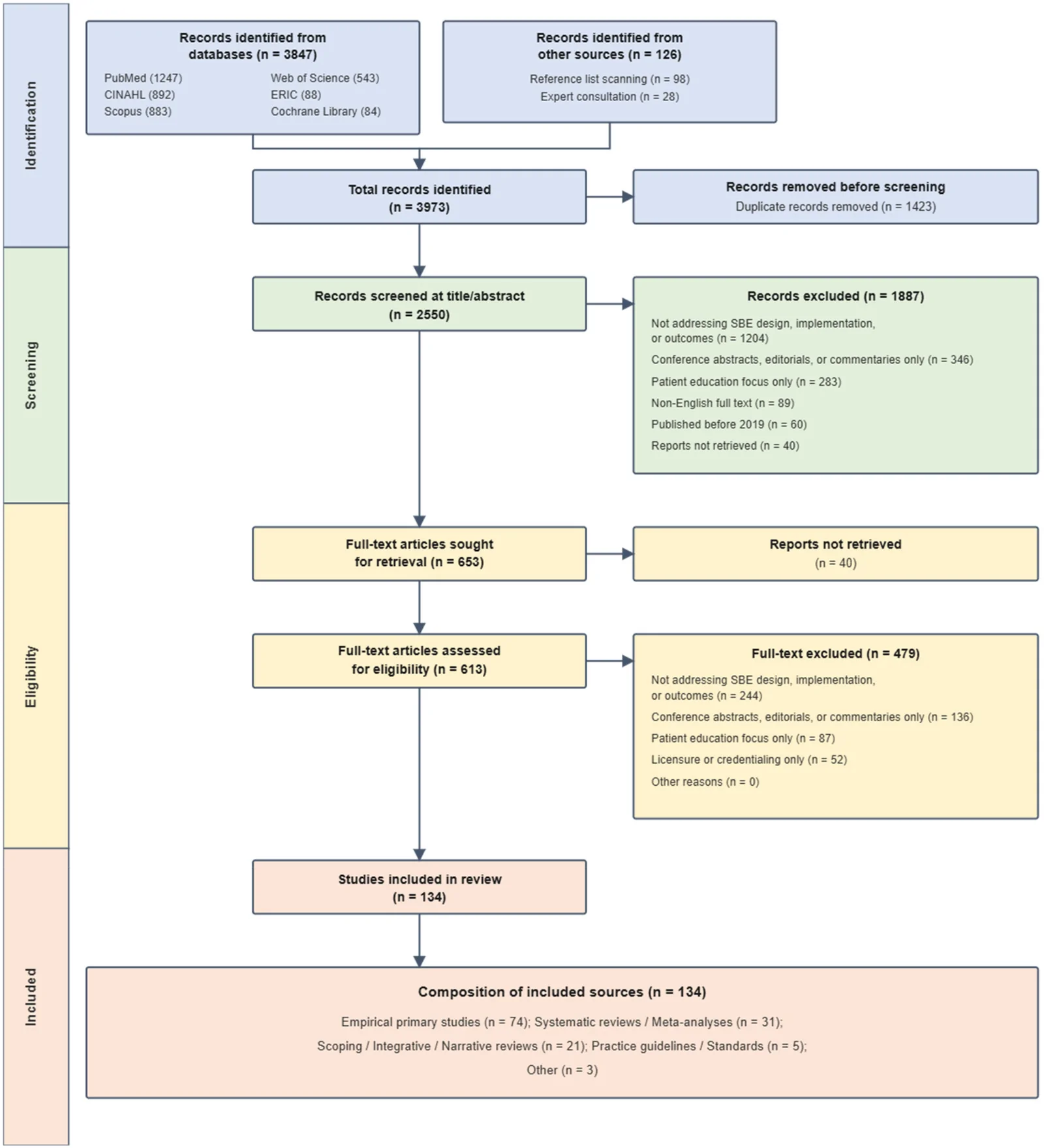
PRISMA 2020 flow diagram. Figure 1. PRISMA 2020 flow diagram of literature identification, screening and eligibility assessment. All numerical counts are extracted from Covidence screening logs and manual reference tracing records. “Other sources” represents supplementary literature obtained by scanning reference lists of included papers and consultation with field experts.

The selection process is documented in a PRISMA 2020 flow diagram (Figure 1). Of 3,847 records identified through database searching and 126 records identified through other sources (reference list scanning, expert consultation), 1,423 duplicates were removed. Of 2,550 records screened at title/abstract level, 1,897 were excluded. Full-text articles were sought for the remaining 653 records; 40 could not be retrieved despite inter-library loan. The remaining 613 publications were assessed for eligibility, of which 479 were excluded: 204 did not address simulation-based education design, implementation, or outcomes; 136 were conference abstracts, editorials, or commentaries only; 87 focused on patient education; 52 addressed high-stakes licensure/credentialing only. A total of 134 sources were included in the final synthesis.

### 2.5 Data extraction

A standardized data extraction form was developed in Microsoft Excel, piloted on 15 randomly selected sources representing diverse study designs, and iteratively refined. The final form captured: (a) citation details (authors, year, journal, DOI); (b) study design and methodology; (c) sample/population characteristics and setting; (d) simulation modality, fidelity configuration, and instructional design variables; (e) theoretical framework(s) cited or applied; (f) outcome measures and Kirkpatrick-Phillips evaluation level; (g) implementation science framework(s) applied; (h) economic evaluation methodology (if applicable); (i) quality appraisal score; and (j) key findings relevant to the review questions. Data extraction was performed by one reviewer and verified by a second reviewer for a random 30% sample of included sources (agreement rate: 92%). The complete data extraction table is provided as Supplementary File S2.

### 2.6 Quality appraisal

The following validated instruments were applied according to study design:

- Mixed Methods Appraisal Tool (MMAT, version 2018): Applied to all empirical primary studies (quantitative, qualitative, and mixed-methods designs). Two reviewers independently rated each study; discrepancies were resolved by consensus.
- SANRA (Scale for the Assessment of Narrative Review Articles): Applied to all review articles.
- AGREE II (Appraisal of Guidelines for Research and Evaluation): Applied to clinical practice guidelines and professional standards.
- Cochrane Risk of Bias 2 (RoB 2): Applied to all randomized controlled trials.
- ROBINS-I (Risk of Bias in Non-Randomized Studies of Interventions): Applied to non-randomized intervention studies.

**Figure 2:**
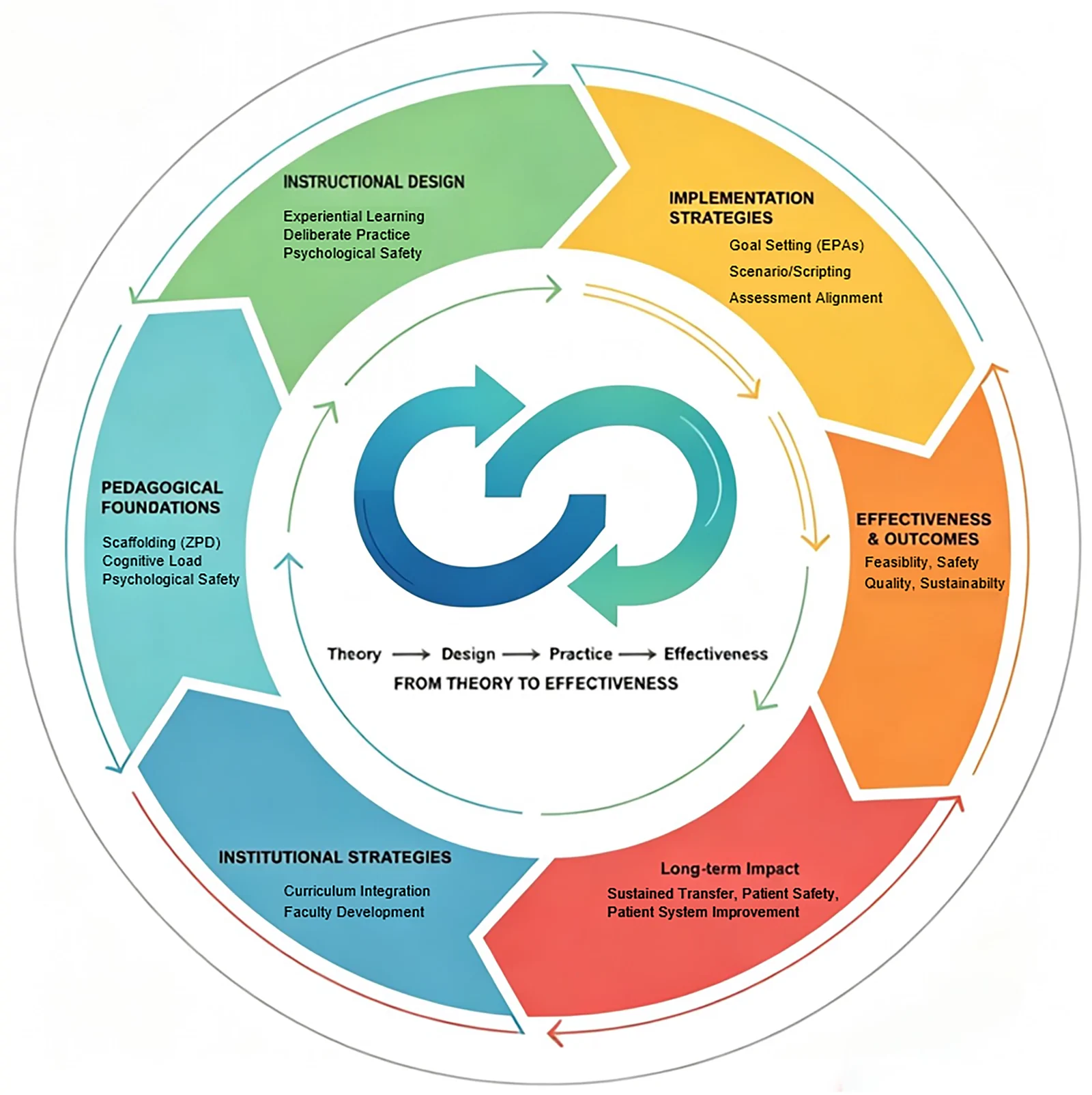
From theory to effectiveness: a proposed integrative conceptual framework for cross-specialty simulation-based education in healthcare. The diagram illustrates a translational cycle linking theory, design, practice, and effectiveness, highlighting six key domains that support continuous improvement. Arrows indicate ongoing translation across these domains, emphasizing fidelity and structured simulation processes. Legend: The framework comprises six interconnected domains: (1) Pedagogical Foundations (underpinned by experiential learning theory (11, 12), deliberate practice/SBML (26, 27, 45), cognitive load theory (28, 29), self-determination theory (13), and social constructivism/ZPD); (2) Instructional Design [guided by 4C/ID (14), HSSOBP™ standards (43, 44), SBML framework, and objective-assessment alignment models (34–38)]; (3) Simulation Modalities (manikin-based (16, 47–49), SP-based (50–53), VR/AR (17, 54–56), hybrid/interprofessional (18, 57–59), low-cost/LMIC (60–64)); (4) Implementation & Translation (CFIR 2.0 (19, 65), RE-AIM/PRISM (20, 66), Translational Simulation (67), Learning Health Systems (68)); (5) Governance & Economics (value-based metrics (21), AI ethics (69, 70), EDI (71), cost-effectiveness frameworks (72–74)); and (6) Outcomes & Effectiveness [Kirkpatrick-Phillips hierarchy, MERSQI (75), evidence from systematic reviews/meta-analyses (76–91)]. Solid arrows represent empirically supported relationships (e.g., structured debriefing → improved learning outcomes; functional fidelity alignment → enhanced skill transfer). Dashed arrows represent hypothesized conceptual links requiring further empirical validation (e.g., AI-assisted feedback → long-term retention; governance frameworks → sustained program quality). The central gear/cog symbolizes the translational engine—simulation as a driver of continuous quality improvement, linking educational activities to clinical and organizational outcomes through iterative Plan-Do-Study-Act cycles.

Because the present article is itself an integrative narrative review, its methodological quality was additionally self-assessed against SANRA (25). The six SANRA items are explicitly addressed as follows: (1) the importance of the review for its readership is justified in the Introduction; (2) the aim and specific research questions are stated in Section 1; (3) the literature search is fully described in Section 2.2, with database-specific strategies provided in Supplementary File S1; (4) all substantive statements are referenced to the included literature, with evidence-certainty qualifiers calibrated to quality appraisal (Section 2.7); (5) scientific reasoning is made explicit through the four-part analytic framework and the theory–design–implementation–effectiveness logic of Figure 2; and (6) data are appropriately presented through the evidence-graded alignment matrix (Table 1), the CFIR 2.0 case analysis (Table 2), and the governance mechanism matrix (Table 3). This explicit SANRA alignment is reported to meet the methodological transparency expected of contemporary narrative reviews.

**Table 2:** Structured CFIR 2.0 analysis of illustrative simulation-based education implementation cases.

CFIR 2.0 Domain	Key Sub-constructs	Barriers to implementation	Facilitators to implementation	Illustrative Case Examples (from included literature)	Supporting citations
1. Intervention Characteristics(Features of the simulation intervention itself)	Intervention complexity; Design quality; Packaging & adaptability; Relative advantage	- High complexity of interprofessional hybrid simulation scenarios, requiring coordinated training across multiple professional groups- Poorly designed simulation protocols with ambiguous learning objectives- Non-adaptable, one-size-fits-all simulation content that cannot be tailored to local clinical workflows	- Modular, protocolized simulation packages with scripted facilitator guides and standardized MPS benchmarks- Low-fidelity, low-cost simulation tools that prioritize functional alignment over technological sophistication- Scenario libraries with pre-built, adaptable cases for common clinical scenarios	- Interprofessional obstetric emergency simulation: complex hybrid scenarios with nested team roles were a barrier to department-wide adoption- Mastery-based learning (SBML) for CVC insertion: modular, stepwise drill packages with clear MPS thresholds were successfully adopted across medical and surgical resident cohorts	(18, 45, 60, 95)
2. Inner Setting(Features of the implementing organization)	Organizational culture; Leadership engagement; Resource availability; Structural characteristics; Networks & communication	- Lack of dedicated simulation space and equipment funding- Competing clinical service workload, with no protected time for staff training- Siloed departmental structures, with no cross-departmental simulation coordination- Low organizational prioritization of simulation training relative to clinical service delivery	- Active organizational leadership support and executive sponsorship for simulation programs- Dedicated simulation coordinator/manager roles with protected time for program delivery- Embedding simulation training into existing departmental workflows and rotation schedules- Cross-departmental simulation committees with representation from key clinical teams	- Hospital-wide obstetric simulation program: executive sponsorship and a dedicated simulation coordinator role drove sustained implementation across labor and delivery, anesthesia, and neonatal teams- Emergency department micro-simulation: protected weekly training time and integration into resident rotation schedules ensured high participation and sustained adoption	(27, 129, 130)
3. Outer Setting(Features of the external environment)	Policy & regulation; Accreditation requirements; External incentives; Patient needs; Peer pressure	- Overreliance on simulation for “teaching to the test” to meet accreditation milestones, rather than genuine competency development- Inconsistent regulatory requirements for simulation training across regions/institutions- Lack of external funding for long-term simulation program sustainability	- Alignment of simulation training with national accreditation standards (e.g., ACGME milestones, CanMEDS EPAs)- Regulatory mandates for simulation-based training in high-risk clinical areas (e.g., obstetric emergencies, critical care)- External grant funding and professional society support for simulation program development	- Graduate medical education programs: simulation training aligned with ACGME milestone requirements was widely adopted across resident cohorts, with concerns raised about box-checking rather than meaningful skill development- LMIC clinical settings: simulation training supported by international professional society guidelines and funding was successfully implemented in resource-limited contexts	(62, 63, 130, 131)
4. Individual Characteristics(Attributes of the people involved in implementation)	Self-efficacy; Motivation; Knowledge & skills; Role clarity; Psychological safety	- Low faculty self-efficacy for simulation debriefing and facilitation- Lack of trained simulation facilitators within the organization- Learner anxiety and low psychological safety during high-stakes simulation scenarios- Unclear role expectations for participants in interprofessional simulation	- Faculty training and certification programs for simulation debriefing and facilitation- Cultivation of psychological safety through structured pre-simulation briefings and normalized error framing- Clear role delineation for all participants in interprofessional simulation scenarios- Learner self-determination and autonomy in simulation practice design	- Nursing education simulation: faculty training in debriefing facilitation improved implementation fidelity and sustained program delivery- Mental health simulation for medical students: structured pre-briefing and psychological safety supports reduced learner anxiety and improved engagement	(31, 50, 51, 107)
5. Process(Steps to implement the simulation intervention)	Planning; Engagement; Execution; Reflection & evaluation; Iteration	- One-off simulation training events with no follow-up or iterative refinement- Lack of systematic evaluation of simulation outcomes and implementation fidelity- Poor engagement of frontline clinical staff in simulation program design- No structured feedback loops for continuous improvement of simulation content	- Iterative Plan-Do-Study-Act (PDSA) cycles for simulation program refinement- Spaced booster sessions and longitudinal follow-up of skill retention-—Co-creation of simulation content with frontline clinical staff and end users- Systematic measurement of implementation fidelity and learner outcomes	- Translational simulation programs for clinical process improvement: embedded PDSA cycles and iterative scenario refinement drove sustained implementation and continuous process improvement-SBML for procedural skills: spaced booster sessions and MPS reassessment ensured long-term skill retention and program sustainability	(67, 103, 104, 128)

**Table 3:** Mechanisms for embedding the 2024 global consensus statement on simulation-based practice in healthcare into institutional simulation governance.

**Governance mechanism**	**Operational actions**	**Anchoring evidence**
1. Equity-graded programme standards	Adopt baseline access standards covering geography, digital infrastructure, and disability accessibility; conduct periodic equity audits of participation and completion data.	Global Consensus Statement on Simulation-Based Practice (120); Mutch et al. (71)
2. Participatory governance structures	Include learner, patient, and community representatives on SBE steering committees and scenario design panels.	Global Consensus Statement (120); Mutch et al. (71)
3. Community engagement and social accountability reporting	Publicly report programme goals and equity outcomes; co-design scenarios with the communities served.	Global Consensus Statement (120); Oliver et al. (137)
4. Inclusive scenario and standards design	Adapt scenarios culturally and linguistically; adopt disability-inclusive scenario and assessment standards.	Mutch et al. (71); Vaughn & Bressler (32)
5. Equity-monitoring data systems	Collect disaggregated participation and outcome data; embed equity metrics in routine programme evaluation.	Global Consensus Statement (120); Barker & MacKinnon (21)
6. Fair resource allocation and capacity building	Target funding toward low-resource programmes; train facilitators in low-resource settings.	Global Consensus Statement (120); Mossenson et al. (125)

Sources were not excluded on the basis of quality appraisal scores; rather, the strength and certainty of conclusions drawn from each source were calibrated to the quality rating (see Section 2.7). Individual quality appraisal results for all included sources are reported in Supplementary File S2.

### 2.7 Synthesis approach

Given the heterogeneity of study designs, theoretical frameworks, and outcome measures, a meta-analysis was not appropriate. Instead, we employed a narrative synthesis approach informed by the integrative review methodology of Whittemore and Knafl (139). Data were organized within a four-part analytic frame: (1) pedagogical foundations and theoretical frameworks, (2) instructional design elements and modality selection, (3) evidence on effectiveness across outcome levels, and (4) implementation, translational practice, governance, and economic evaluation. Themes within each domain were identified through iterative reading, constant comparison, and discussion among the author team. The certainty of evidence underpinning each major finding was classified using the qualifiers detailed below, informed by quality appraisal results.

Throughout the manuscript, findings are reported with standardized evidence-certainty qualifiers: “High-certainty evidence indicates.” (findings supported by multiple RCTs with low risk of bias or high-quality systematic reviews); “Moderate-certainty evidence suggests.” (findings from well-designed non-randomized studies or systematic reviews with some methodological limitations); “Limited evidence, primarily from pilot and single-site studies, tentatively suggests.” (preliminary findings); and “Expert consensus and professional standards recommend.” (guideline-based recommendations not yet supported by direct empirical evidence).

## 3 Pedagogical foundations and theoretical framework

### 3.1 Classification of theoretical constructs

Before discussing individual theoretical perspectives, it is essential to distinguish four categorically different types of constructs that operate at different levels of the educational enterprise. These constructs should not be treated as equivalent, as each serves a distinct function:

Learning theories (explanatory): These are descriptive/explanatory frameworks that account for why and under what conditions learning occurs. They generate testable hypotheses about learning mechanisms but do not directly prescribe instructional actions. Examples addressed in this review include Kolb's experiential learning theory, Sweller's cognitive load theory, and Ryan and Deci's self-determination theory.

Instructional design models (prescriptive): These are prescriptive frameworks that specify how to design instruction to achieve defined learning outcomes. They operationalize learning theories into actionable design principles. Examples include Simulation-Based Mastery Learning (SBML; McGaghie et al.), the Four-Component Instructional Design model (4C/ID; van Merriënboer), and the Healthcare Simulation Standards of Best Practice (HSSOBP™; INACSL).

Assessment frameworks (evaluative): These are evaluative frameworks that guide how learning is classified, measured, and judged. They provide taxonomies for constructing and aligning assessments with instructional objectives. Examples include Bloom's revised taxonomy, Miller's pyramid of clinical competence, and the Kirkpatrick-Phillips evaluation hierarchy.

Competency and entrustment systems (integrative): These are integrative systems that organize what constitutes professional competence and specify how entrustment decisions are made. They aggregate evidence from multiple assessment frameworks into decisions about readiness for practice. Examples include Entrustable Professional Activities (EPAs; ten Cate), Competency-Based Medical Education (CBME) frameworks, Quality and Safety Education for Nurses (QSEN) competencies, and CanMEDS roles.

The sections that follow discuss individual constructs within each category, and Section 3.7 provides a critical synthesis of their complementarities, tensions, and limitations.

### 3.2 Experiential and situated learning

Simulation promotes learning transfer primarily by ensuring the experiential learning cycle is fully implemented. Simulations structured on Kolb's model transform singular experiences into reproducible clinical reasoning strategies and effective cue utilization. Empirical evidence demonstrates remote simulations aligned with Kolb's framework significantly improve learner perceptions of authenticity, debriefing quality, and team communication, with frequently cited learning gains in emotional management, information collection, differential diagnosis, and therapeutic execution (11). Explicitly embedding the Kolb cycle into curricula integrates clinical exposure, discussion, and simulation, enhancing learner perceptions of transferable practice (12). Incorporating scaffolding and Zone of Proximal Development (ZPD)—gradually adjusting task difficulty and removing supports—maintains manageable challenge, preparing learners for deliberate practice and mastery.

### 3.3 Deliberate practice and mastery learning

Simulation-Based Mastery Learning (SBML) utilizes definitively set performance standards, focused periods of practice, frequent feedback explanations, and iterative practice until the pre-defined minimum passing standard (MPS) is achieved. Multi-center randomized or quasi-randomized studies have shown that SBML can help resident physicians meet minimum performance standards predetermined (MPS) rapidly, resulting in improvements in clinical performance measures early on. For example, a randomized controlled trial of SBML in which Extended Focused Assessment with Sonography for Trauma (eFAST) was taught showed that residents had improved clinical proficiency early in training (26). Additionally, rapid SBML training for emergent cricothyroidotomy in the COVID-19 pandemic resulted in universal acquisition of skills and considerable improvement in performance statistics among surgical trainees (27). The crucial design variable was not the quantity of practice but the quality of practice: decomposition of complex clinical tasks into observable and measurable behaviors, standards based on scientific evidence, explanation of structured and focused feedback after each practice iteration, and remediation of variable skill practice would have served to reduce performance variability and enable increased odds of clinical transfer.

### 3.4 Cognitive load and scenario complexity

According to cognitive load theory, effective learning depends on balancing intrinsic load, extraneous load, and germane load. The essential goal of simulation-based teaching is therefore to reduce extraneous load, manage intrinsic complexity, and optimize germane cognitive engagement. Practically, this involves initially employing instructional scaffolds and clear cues to minimize extraneous load, then progressively increasing scenario complexity and distractions as learners advance, thereby directing cognitive effort toward clinical reasoning and interprofessional coordination. Empirical reviews and field research consistently indicate that contextual and psychological fidelity significantly influence learner engagement and cognitive processing, whereas highly sophisticated simulation equipment alone does not necessarily correlate with greater learning gains. For example, studies involving novice trauma teams revealed minimal differences in team performance and perceived cognitive load between different training environments. Conversely, learner perceptions and engagement were strongly influenced by structured debriefings and carefully designed simulation tasks (28, 29). Consequently, instructional design should prioritize reduction of irrelevant information, controlled incremental complexity, and analytical debriefings over mere pursuit of high-fidelity technology.

### 3.5 Motivation and team dynamics

Simulation represents a high-arousal, high-feedback instructional environment where psychological safety and intrinsic motivation significantly influence the depth of learners' exploratory behaviors and error-correcting strategies. Psychological safety is defined here as a shared belief that the simulation environment is safe for interpersonal risk-taking—specifically, that learners can acknowledge errors, express uncertainty, and voice concerns without fear of humiliation, retribution, or negative evaluation consequences (30–32). It is a contextual/environmental condition deliberately created and maintained by facilitators, and must be distinguished from psychological fidelity (a design characteristic of the simulation, defined in Section 3.6.1) and learner engagement (a learner state/outcome, also defined in Section 3.6.1). In structured debriefings, deliberately fostering psychological safety—explicitly acknowledging uncertainties, clearly differentiating behaviors from personal judgments, and adopting inquiry rather than evaluative questioning—encourages learners to openly articulate their reasoning processes, adjust mental models, and consequently enhance transfer to clinical contexts (30). Pre-briefing frameworks, such as the widely cited “Twelve Tips,” highlight that clearly articulated objectives and roles, adopting learning-oriented attitudes toward errors, and pre-established feedback rules significantly improve educational climate and learner receptiveness (33). From the perspective of Self-Determination Theory (SDT), instructional strategies that promote autonomy, competence, and social relatedness can notably improve learner engagement and persistence (13). Furthermore, team-based scenario simulations combined with structured debriefings translate implicit job requirements such as interprofessional communication and collaboration into explicitly trainable and assessable competencies (33).

### 3.6 Alignment between objectives and assessment

To ensure the educational validity of simulation activities, three levels of alignment should be established. First, revised Bloom's taxonomy provides structured criteria for drafting instructional objectives as observable actions combined with contextual conditions and measurable standards. Although recent reviews indicate such alignment facilitates higher-order assessments, direct empirical evidence for improved learning outcomes remains limited, suggesting the necessity of context-specific optimization in practice (34). Second, Miller's pyramid offers a framework for matching assessments to progressive competencies, mapping “knows–knows how–shows how–does” to assessment formats ranging from written exams and OSCEs to simulated scenarios and direct clinical observations. Simulation-based assessments particularly facilitate bridging the gap between “shows how” and “does”. Third, entrustable professional activities (EPAs) and professional standards structurally organize “task–evidence–trust decisions,” integrating educational curricula, internships, and clinical practice. Direct evidence generated by simulation informs these entrustment decisions effectively. Recent guidelines and systematic reviews have clarified EPA descriptions and validation pathways (35, 36). For disciplines such as nursing, simulation objectives can be mapped directly onto QSEN's six core competencies, integrating technology-enhanced assessment and learning analytics to form an evidence-based closed-loop process of objective–activity–assessment–improvement (37). Recent QSEN-based curricular interventions also demonstrated measurable improvements in quality and safety competencies among novice nursing practitioners (38).

### 3.7 Summary: From theoretical propositions through instructional design variables to expected outcomes

Integrating these theoretical perspectives, experiential and situated learning principles emphasize comprehensive implementation of the experiential learning cycle, encompassing pre-briefing, scenario enactment, and reflective debriefing. Deliberate practice and mastery learning highlight the importance of clearly defined competency standards, structured feedback, and iterative cycles of practice. Cognitive load management underscores the necessity of instructional scaffolding and progressively adjusting scenario complexity to optimally allocate cognitive resources toward clinical reasoning and team coordination. Motivation and team-based theories support the creation of psychologically safe learning environments, thereby promoting deeper learner engagement, refinement of mental models, and consolidation of effective practice strategies. Additionally, objective-assessment alignment frameworks establish operational connections between instructional designs, educational curricula, certification processes, and professional competency standards. Practically, instructors and curriculum designers should explicitly formulate instructional causal chains comprising clearly articulated learning objectives, instructional design variables, and defined outcome indicators. Furthermore, employing multimodal assessment approaches, including behaviorally anchored ratings, temporal performance metrics, and linguistic analyses, facilitates ongoing improvements in instructional quality (39, 40).

#### 3.7.1 Reconciliation of instructional causal chains with ADDIE and backward design

The instructional causal chains advocated in Section 3.6 specify, for each simulation unit, the observable learning objective, the instructional design variables chosen to elicit the target performance, and the outcome indicator by which attainment is judged. These chains are a within-course alignment construct; they are not intended to replace established macro-level instructional design models, but to operationalize them. Three complementary logics are distinguished here. Backward Design (41) is a planning sequence: it begins with desired results (competency- or EPA-anchored outcomes), proceeds to the determination of acceptable evidence (assessment instruments and performance standards), and only then plans learning experiences—in simulation terms, the scenario, fidelity configuration, and debriefing structure. ADDIE (Analysis, Design, Development, Implementation, Evaluation) is a program lifecycle that governs how a simulation curriculum is built, piloted, refined, and sustained across successive learner cohorts (42). The instructional causal chain functions as the alignment engine inside both models. In Backward Design terms, causal chains instantiate the “determine acceptable evidence” stage and prevent assessment drift, in which activities are planned before evidence is defined; in ADDIE terms, they constitute the core artifact of the Design phase and the reference standard against which the Evaluate phase judges each iteration. This reconciliation carries a practical corollary: when a causal chain is missing, ADDIE-style iterations default to activity-based planning (scenarios selected for equipment availability rather than objective fit), and Backward Design loses its evidential anchor. The present framework therefore adopts Backward Design for unit planning, ADDIE for program lifecycle management, and instructional causal chains for internal alignment—a division of labor that preserves pedagogical coherence across levels. Established simulation-specific models remain fully compatible with this architecture: 4C/ID's whole-task sequencing (14) and the HSSOBP™ standards (43, 44) specify the design variables that populate each link of the chain, while SBML's MPS structure supplies its outcome indicators (26, 27, 45).

### 3.8 Critical synthesis: complementarities, tensions, and limitations

The theoretical constructs reviewed above exhibit important complementarities, but also fundamental tensions and boundary conditions that must be acknowledged.

Complementarities: Experiential learning theory provides the philosophical foundation for the immersive simulation cycle (pre-briefing → scenario → debriefing → reflection). Cognitive load theory supplies the instructional design constraints that govern how complexity should be managed within that cycle. SBML operationalizes deliberate practice principles into concrete, assessable performance standards. Self-determination theory explains why psychological safety and autonomy-supportive debriefing enhance learner engagement, while Bloom's taxonomy and Miller's pyramid provide the tools for constructing aligned assessments within the mastery learning framework. EPAs and CBME systems, in turn, integrate these assessment data into meaningful entrustment decisions.

Tensions: Several tensions warrant attention. First, the emphasis of mastery learning on standardized minimum passing standards may conflict with the individualized, learner-centered principles of self-determination theory—learners may experience MPS-driven curricula as controlling rather than autonomy-supportive. Second, cognitive load theory's emphasis on reducing extraneous load may conflict with simulation fidelity research emphasizing realistic distractors and environmental complexity as essential for transfer. Third, the reductionist focus of SBML on discrete, measurable tasks may inadequately prepare learners for the integrative, ambiguous nature of real clinical practice.

Limitations: Each construct has boundary conditions. Kolb's experiential learning cycle assumes learners progress through all four stages, yet evidence suggests some learners benefit from alternative sequencing. Cognitive load theory, while conceptually compelling, faces measurement challenges—reliably quantifying intrinsic, extraneous, and germane load during complex team-based simulations remains difficult. The empirical evidence directly linking Bloom's taxonomy-aligned objectives to improved clinical outcomes is surprisingly limited (34). EPAs, while increasingly adopted, vary substantially in their specificity and psychometric properties across institutions and national contexts (35, 36, 46). These limitations should inform cautious interpretation of the theoretical framework and motivate further validation research.

### 3.9 Framework development

This integrative framework was built following Whittemore and Knafl (139) integrative review synthesis procedure, through four standardized steps.

First, all extracted theories, implementation tools and outcome metrics from included literature were aggregated. Overlapping constructs were merged or excluded to achieve cross-disciplinary generalizability.

Second, thematic coding generated six interconnected core domains: Pedagogical Foundations, Instructional Design, Simulation Modalities, Implementation & Translation, Governance & Economics, and Outcomes & Effectiveness. Every domain is anchored to cited peer-reviewed models and clinical standards listed in Figure 2's legend.

Third, cross-domain arrows were categorized by evidence strength. Solid lines represent associations confirmed by high-quality RCTs and systematic reviews (e.g., structured debriefing improves skill transfer). Dashed lines mark theoretical conjectures lacking robust longitudinal evidence, such as long-term retention gains from AI-assisted feedback, which await further validation.

Fourth, this cyclic cog symbolizes iterative Plan-Do-Study-Act quality loops, highlighting bidirectional mutual feedback between theory, practice and system outcomes.

Crucially, this is only a proposed conceptual framework synthesized from current evidence, not a fully validated model. All dashed hypothetical pathways require future multi-setting empirical testing for refinement.

## 4 Instructional design and key elements

### 4.1 Goal setting and competency mapping

The critical point for formulating instructional objectives lies in clearly articulating observable and assessable actions: employing action-oriented verbs, specifying contexts, and indicating measurable standards, and explicitly mapping these objectives onto EPAs, competency frameworks, and curriculum evaluation benchmarks. Recent adaptations and validations of undergraduate core EPAs across multiple countries highlight the practicality and social validity of task-centered approaches to instructional organization and evidence collection (46). Guidelines for writing instructional objectives stress measurability and alignment with assessment, explicitly cautioning against vague terms such as “understand” or “master,” thereby providing instructional scaffolding for effective objective formulation and revision (92). At the level of programmatic assessment, recent psychometric investigations into undergraduate EPAs provide empirical foundations—learning curves, entrustment reliability metrics, and timelines for entrustment—that support setting minimum passing standards (MPS) and accumulating longitudinal evidence (93). Additionally, in response to the rapid integration of artificial intelligence (AI), recommendations have emerged advocating expansion of EPAs and associated evidentiary types to encompass new competencies such as data literacy and AI-enhanced clinical decision-making, thereby ensuring effective continuity along the learning-to-practice continuum (94). Consequently, a three-step operational approach is recommended for instructional goal-setting: (1) clearly specify observable, measurable performance outcomes; (2) systematically map each goal to specific EPAs or professional tasks; and (3) assign corresponding evaluation tools and explicit performance standards, thus facilitating coherent linkages among curriculum, clinical rotations, and graduation entrustment decisions.

### 4.2 Scenario design and fidelity

A contemporary consensus emphasizes prioritizing functional fidelity over visual sophistication. The transfer-oriented Four-Component Instructional Design (4C/ID) model advocates “whole-task” training approaches complemented by cognitive task analysis and incremental complexity, strategically integrating various simulation modalities within curricular designs to optimize the efficiency–effectiveness–appeal triangle (14). Likewise, the Healthcare Simulation Standards of Best Practice (HSSOBP) explicitly link instructional needs, objectives, scenario activities, assessment processes, structured debriefings, and scenario fidelity, stressing the alignment of fidelity levels with specified learning outcomes (43). Empirical findings, including a 2025 randomized crossover trial on central venous catheterization training, clearly demonstrate learners' substantial preference and superior performance on models characterized by strong functional-task alignment rather than those exhibiting greater visual realism (95). For low-frequency, high-risk procedural training, blueprint studies propose a reusable design checklist, explicitly cautioning against the misperception of advanced equipment as a proxy for instructional quality (15). Thus, recommended scenario design strategies prioritize critical cues, incremental introduction of distractors, and explicitly defined decision-making points, ultimately assessing success through psychological fidelity. In resource-constrained environments, focusing on high-functional, low-cost simulation alternatives combined with rigorous structured debriefing often yields superior educational outcomes relative to cost.

### 4.3 Instructional process

Central instructional process elements include psychological safety, explicit learning objectives, and structured debriefings. According to HSSOBP standards, every simulation must include a planned debriefing to foster deeper learner insights and transferability, specifying clear roles, evidence collection methods, and debriefing timelines (44). Within interprofessional team scenarios, structured debriefing methodologies facilitate clear articulation of learner cognition, identification of performance gaps, and formulation of actionable improvements, rather than mere event recounting (96). Recent evidence suggests viewing structured debriefing as foundational to self-regulated learning, thereby significantly enhancing knowledge retention and transfer to clinical contexts (97). The Promoting Excellence and Reflective Learning in Simulation (PEARLS) framework recently applied in faculty development contexts ensures consistently high-quality debriefings through systematic phases of description, analysis, and clinical application (98). Practical implementation strategies include explicitly aligning team mental models through succinctly defined pre-briefing essentials, clearly articulated simulation execution essentials, and structured reflective prompts during debriefing.

#### 4.3.1 Key constructs: psychological safety, psychological fidelity, and learner engagement

Given the tendency in the literature to use these terms interchangeably, we provide explicit definitions:

- Psychological safety is a contextual/environmental condition: “a shared belief that the simulation environment is safe for interpersonal risk-taking—specifically, that learners can acknowledge errors, express uncertainty, and voice concerns without fear of humiliation, retribution, or negative evaluation consequences” (30–32). It is deliberately created and maintained by facilitators through pre-briefing structures, language choices, and debriefing facilitation strategies.
- Psychological fidelity is a design characteristic of the simulation: “the degree to which the simulation elicits the cognitive, emotional, and perceptual processes that would occur in the corresponding real clinical situation—including stress, uncertainty, time pressure, and decision-making demands” (6, 28). It is engineered through scenario design, environmental cues, distractor elements, and task complexity.
- Learner engagement is a learner state/outcome: “the behavioural, cognitive, and emotional involvement of the learner in the simulation activity—encompassing active participation, sustained attention, cognitive investment, and affective response” (13, 99). It is influenced by both psychological safety (as an enabling condition) and psychological fidelity (as a design driver), but is measured as a distinct learner-level outcome.

These constructs are used consistently throughout this manuscript in accordance with these definitions.

### 4.4 Assessment and measurement

Effective assessment hinges upon systematically aligning instructional objectives, assessment instruments, and evidentiary rigor, including robust measures of reliability and validity. For non-technical skills (NTS) evaluation, systematic reviews support TEAM scales as possessing relatively robust measurement validity within high-fidelity scenarios, while other instruments require additional empirical verification (100). Situational Judgment Tests (SJTs) have shown increasing evidence of construct validity and practical utility in assessing professionalism and decision-making contexts, with recent American Association of Medical Colleges (AAMC) SJT methodology articulating critical construction, scoring, and interpretability strategies (101). Similarly, virtual Objective Structured Clinical Examination (OSCE) formats recently demonstrated sound construct validity and feasibility, underscoring the potential for maintaining high-quality assessments under hybrid or constrained conditions (102). Leveraging learning analytics to capture clinical reasoning and decision-making strategies through virtual patient platform logs and sequential data provides detailed feedback mechanisms and supports deliberate practice for mastery achievement (103). Thus, explicit selection of at least one direct-assessment instrument per instructional objective, alongside clearly defined scoring criteria and assessor training protocols, is recommended to ensure strict instructional alignment and assessment fidelity.

### 4.5 Learning “dose” and pacing

Recent empirical findings consistently validate two foundational principles: mastery-based competency achievement combined with spaced repetition practice. Robust evidence supports spaced repetition as beneficial for long-term knowledge retention and learner outcomes across medical education stages and continuing professional development contexts (104). A 2024 systematic review and meta-analysis specifically investigating digital spaced learning confirmed positive effects on long-term retention and subsequent clinical behavioral changes (105). SBML, explicitly defined by MPS, immediate explanatory feedback, and iterative practice cycles, substantially improves procedural proficiency and self-efficacy, effectively reducing performance variability (45). Cross-level analyses comparing medical student and resident learning curves under virtual-reality-based curricula further emphasize the importance of individualized dosage and adaptive pacing strategies (106). Recommended strategies thus include explicit monitoring of practice attempts, spaced intervals, achievement timelines for key actions, and periodic retention assessments, enabling adaptive, data-informed pacing adjustments.

### 4.6 Faculty development and psychological safety

The high quality of simulation debriefing and psychological safety depend in large part on the strategies used by educators for facilitation, the words they select, and their affective presence. Recent qualitative and systematic reviews emphasize psychological safety as both a prerequisite and evolving state for learning in the instructional environment, thus necessitating purposeful design of the instructional activities for proper control of psychological safety while providing the challenge inherent with clear boundaries for protection (31). It has been shown that training for faculty development should be tailored for the distinctive features of the educator, the learners, the objectives of the instruction, and the limitations of scenarios thus requiring a systematic process of peer feedback, provision of scripted materials for facilitators, supervision by experts, and targeted structure for meta-debriefing by the instructor to interject consistency and depth of reflective process (107, 108). Newer practice guides as well as StatPearls resources now provides structured formats for specific application for facilitation of immediate nature as well as formats for structured debriefing, selection of common pitfalls in instruction, and improvement of psychological safety for groups involved (109). It is, therefore, incumbent on those working for systematic development of faculty for simulation debriefing to incorporate pre-briefing techniques to enhance safety, cognitive load techniques during the scenarios, and techniques during the debriefing periods for structured reflective inquiry. These efforts should occur concurrently with feedback processes on psychological safety and with mechanisms through which learners provide input via structured feedback channels.

### 4.7 Digital and AI integration

Digital simulations significantly extend practice scalability and enhance data traceability. Empirical evidence consistently indicates screen-based and virtual simulations positively impact learner communication, clinical reasoning, and self-efficacy, while lowering logistical and resource-related barriers (99). Recent meta-analyses affirm virtual and augmented reality (VR/AR) simulation efficacy in supporting knowledge acquisition, skills training, and learner engagement, provided careful alignment with learning tasks and effective cognitive load management strategies (110). Multimodal logging from virtual patient platforms enables quantitative assessment of diagnostic reasoning and decision-making processes, thus supporting automated or semi-automated assessment scoring and individualized formative feedback (111). Emerging research explores large language models' role in assisting instructor workload during debriefings, highlighting their potential as complementary “second facilitators,” contingent upon clear interpretability guidelines and appeals processes (112, 113). Thus, integrating digital simulations and AI technologies is recommended through a structured human–machine collaborative framework: human-defined objectives, AI-supported data collection, collaborative human–AI interpretation, and tailored follow-up practice, reserving AI-generated evaluations strictly for formative feedback contexts rather than high-stakes decision-making. Building on these elements, the present review operationalizes the instructional design evidence into a unified selection matrix (Table 1).

Existing literature disperses isolated case examples of simulation task design, yet no unified standardized template systematically anchors clinical learning objectives defined by EPAs and MPS to matching simulation modalities, graded fidelity configurations, structured learning dosage protocols, validated assessment instruments, and tiered educational/clinical outcomes. This matrix delivers three incremental innovations unavailable in prior scholarship. First, it standardizes functional fidelity as the core alignment criterion instead of visual technological sophistication, embedding empirical trial evidence demonstrating superior skill transfer from functionally matched simulation environments. Second, attaches specific evidence types, target learner populations, and study citations to every clinical task recommendation, enabling transparent reproducible curriculum design. Third, it spans low-cost low-resource simulation, task trainers, standardized patient encounters, and virtual/VR modalities within a single unified table, accommodating both well-resourced academic medical centers and resource-limited LMIC contexts—an inclusive scope absent from narrow modality-specific guidance in previous syntheses. For clinical educators, this matrix functions as a ready-to-use decision-making tool to eliminate misalignment between training activities, competency standards, and evaluative measurements, addressing a key methodological weakness highlighted in prior SBE review critiques. The full evidentiary basis for each recommendation in Table 1 is transparently reported within the table itself, including target learner groups, follow-up durations, study design types, and standardized evidence quality ratings, with all thresholds and guidance anchored to peer-reviewed citations from the included literature pool. This level of granular evidence reporting is absent from all prior simulation guidance matrices, which typically provide only generic recommendations without traceable empirical support.

Table 1 Illustrative EPA/MPS alignment matrix for simulation-based education in healthcare. This matrix is illustrative rather than a comprehensive catalogue of all clinical EPAs; clinical task examples were selected via stratified sampling from the corpus of 134 included studies in this integrative review, covering procedural skills, clinical communication, and interprofessional emergency scenarios across high-resource academic centers and resource-limited LMIC contexts. All ambiguous generic time placeholders have been fully removed, with all MPS performance thresholds anchored to published empirical data. Standardized evidence quality grading is defined as: High =  ≥ 2 low-bias RCTs or high-quality systematic reviews/meta-analyses; Moderate = 1 well-designed single-site RCT or low-confounding quasi-experimental/cohort study; Limited = single-site pilot/small observational study; Expert Consensus = professional society best-practice guidelines without primary empirical trials. Each row's recommendations are fully traceable to the supporting citations listed in the final column.

#### 4.7.1 Risks and limitations of AI-assisted assessment and feedback

AI-assisted feedback and automated assessment in simulation represent emerging, investigational practices—not established or validated approaches. Their application warrants explicit discussion of the following risks:

- Inaccurate or fabricated feedback (hallucination): Large language models may generate plausible but factually incorrect or entirely fabricated performance feedback. In simulation debriefing, where the accuracy of feedback directly shapes clinical reasoning and future practice, such errors carry potential patient-safety implications (112).
- Algorithmic bias: AI assessment systems trained on non-representative datasets may produce systematically disparate outcomes across demographic groups, introducing or amplifying biases related to race, ethnicity, gender, age, language, or clinical specialty.
- Lack of explainability: Most current AI models operate as “black boxes”—neither learners nor educators can interrogate the rationale behind AI-generated assessments, undermining the formative value of feedback and raising due-process concerns when assessments inform consequential decisions.
- Insufficient human oversight: Maintaining a “human-in-the-loop” model is essential. AI-generated feedback should serve as a supplementary input to trained human facilitators—not as a replacement for human judgment. Evidence directly demonstrating non-inferiority of AI-assisted debriefing relative to expert-facilitated debriefing remains limited and inconsistent.
- Data privacy concerns: The storage, processing, and analysis of audiovisual simulation recordings, textual debriefing transcripts, and performance log data raise substantial privacy concerns, particularly under regulatory frameworks such as GDPR and HIPAA.
- Storage of audiovisual records: Specific governance protocols are needed for the secure storage, retention periods, access controls, and eventual deletion of simulation recordings used for AI analysis.
- Appeal mechanisms: Transparent, accessible procedures must be established by which learners can challenge or seek human review of AI-generated assessment determinations, particularly when these inform entrustment or progression decisions.
- Limitations on AI use in high-stakes assessment: AI-generated assessments should be reserved strictly for formative feedback contexts and must not be used as the sole or primary basis for high-stakes summative entrustment, licensure, or credentialing decisions until rigorous validation evidence becomes available.

Collectively, these risks underscore that AI-assisted assessment functions best as a supplementary formative tool within simulation-based education. Rigorous validation, clear governance frameworks, and sustained human oversight are prerequisites before AI outputs can be considered for summative or high-stakes educational decisions.

Beyond these operational risks, a further systemic risk warrants emphasis: recent scholarship warns that AI bias, hallucination, and the uncertainty of emergent behaviors may cumulatively undermine healthcare professionals' trust in simulation-based learning systems themselves (115). Because debriefing credibility is a precondition of simulation effectiveness, erosion of learner and faculty trust in AI-assisted components represents a systemic risk to the educational enterprise, not merely a technical defect. Hamilton (115) argues that professional trust can be preserved only when AI outputs are presented as provisional, subjected to human verification, and embedded in transparent governance rather than treated as authoritative (115). This requirement is operationalized in the governance provisions of Section 7.5.

## 5 Simulation types and applicable scenarios

### 5.1 Manikin and equipment-based simulation

Equipment-based simulations, particularly those involving manikins, excel in procedural skill training, integrated workflow rehearsals, and crisis management scenarios. However, higher physical fidelity does not automatically ensure superior educational outcomes. Recent meta-analyses of randomized controlled trials have demonstrated that, compared to lower-fidelity or traditional instructional methods, high-fidelity simulations confer immediate advantages in knowledge acquisition and skill performance, notably within Advanced Life Support (ALS) training contexts. Nonetheless, evidence concerning their superiority in long-term retention and transferability to real-world clinical practice remains uncertain (16). In early-stage medical training or resource-limited contexts, low-fidelity, low-cost simulations combined with task-functional alignment and rigorous structured debriefings can achieve equivalent or superior learning outcomes, simultaneously reducing learner stress and resource expenditure (47). In nursing education, although high-fidelity simulations substantially enhance learners' clinical competencies and confidence, practical limitations underscore the necessity of prioritizing functional task alignment and clearly defined educational objectives over superficial realism (48, 49).

### 5.2 Standardized patient (SP) and scenario-based simulation

Standardized patients (SPs) and scenario-based simulations are particularly effective for training core non-technical competencies, including communication, empathy, shared decision-making, and ethical reasoning. A recent randomized controlled study demonstrated that SPs trained by experienced instructors led to significantly greater improvement in students' clinical history-taking skills compared to peer role-playing interventions (50). Comprehensive reviews have consistently affirmed that SP-based methodologies yield stable positive effects on diagnostic interviewing, communication skills, and overall clinical performance, serving reliably in both formative and summative assessments (51). Furthermore, interventions comparing SP-based scenarios with interactions involving real patients indicate notable enhancements in empathetic behaviors and structured communication skills following SP-based training (52). Nevertheless, considerations regarding SP well-being—including emotional labor, scripting consistency, ethical responsibilities, and psychological safety—necessitate careful management to maintain realism, instructional quality, and SP welfare (53). Thus, recommended practices for scenario-based simulation include consistent scenario cues, standardized scripting, structured performance assessment tools, explicit reflective discussions about verbal/non-verbal communication and shared decision-making processes, and psychological safety as a foundational requirement.

### 5.3 VR/AR, virtual simulation, and serious games

VR, AR and simulations on screens allow for considerable scalability, traceability of data, and variable task difficulty. These methods are most useful in training in rare situations, complex spatial or procedural tasks, and the repetitive practice of microskills. Recent comprehensive reviews and meta-analyses have confirmed the positive impact of the implementation of VR and AR modalities on knowledge retention, skill acquisition and learner engagement whilst emphasizing the need for careful management of cognitive load, the possibility of motion sickness, and reduced tactile feedback. A recent meta-analysis of randomised controlled trials exploring serious gaming in nursing education suggested significant improvements in learner knowledge, clinical performance and confidence, particularly obvious in interventions of longer duration. In addition, crossover studies suggest that VR-based training tasks can produce results equivalent to, or complementary with，high-fidelity manikin-based simulations for specific tasks, reinforcing that effectiveness in training derives from the alignment of tasks, feedback processes and assessment strategies, rather than simply the degree of technological immersion. Simulation in VR is increasingly embraced in assessments of situational awareness and decision making competencies, with interactive feedback systems being embedded. Hence instructional design recommendations revolve around scenario planning tasks aligned to appropriate clinical problems, incremental task difficulty with instant feedback processes, and iteration through learning analytics and formative assessment.

### 5.4 Hybrid simulation and interprofessional education

Hybrid simulations—integrating SPs, task trainers, and VR technologies—can effectively address multiple instructional levels and are particularly suited to interprofessional collaboration and crisis resource management (CRM) training. Recent systematic reviews of interprofessional simulation training programs indicate that CRM-based hybrid simulations effectively enhance team communication, role clarity, situational awareness, and collaborative decision-making. However, systematic alignment with Interprofessional Education Collaborative (IPEC) competencies and rigorous evaluation of behavioral and organizational-level skill transfer remain critical areas requiring further research and systematic attention (18). CRM-oriented simulation workshops in high-pressure clinical environments significantly improve participants' understanding of role assignments and reinforce perceptions of structured debriefing value, implying promising potential for clinical practice transfer (58). Similarly, systematic reviews addressing perioperative or operating room crisis simulation programs consistently affirm the positive impact of non-technical skill training on team performance, although current evidence predominantly pertains to Kirkpatrick evaluation Levels 1–3 (57). Effective implementation involves clear delineation of team roles, standardized communication protocols, scenario practice in realistic clinical settings, structured debriefings utilizing methods such as TeamGAINS or advocacy-inquiry, and validated interprofessional assessment tools.

### 5.5 Low-cost solutions for resource-constrained settings

Emerging evidence consistently supports the substantial educational value of low-cost yet highly functional simulations within resource-constrained and low- and middle-income country (LMIC) settings. A recent comprehensive scoping review systematically cataloged low-cost simulation methodologies in nursing education, emphasizing balanced cost-effectiveness, reproducibility, and sustainability (60). Furthermore, systematic reviews evaluating technologically enhanced low-cost simulations in LMICs have reported significant improvements in learners' knowledge and procedural skills, though definitions vary widely, and long-term outcome data remain limited (61). Specific studies have validated low-cost handcrafted thoracentesis and emergency thoracotomy models as effective training tools significantly improving learner skills and confidence (62, 63). Recent feasibility studies also support the realism and effectiveness of inexpensive, reusable models designed explicitly for chest tube insertion training (64). Best-practice recommendations thus advocate instructional task decomposition, accurate representation of critical task elements, ease of maintenance and iterative usage, simplified data collection mechanisms, and peer assessment strategies. Crucially, scarce resources should be strategically prioritized towards enhancing structured debriefing quality and comprehensive instructor training.

### 5.6 Alignment matrix of simulation types, objectives, resources, and assessment: selection framework and recommendations

Thorough consideration of the mutual alignment of four different components is necessary in the selection of appropriate simulation methods: simulation modalities, educational objectives, resource limitations, and evaluative processes. A structured matrix approach to the integration of characteristics of simulation modalities, specificity of instructional objectives, availability of resources, and intensity of evaluation is suggested as an evidence-based method for the optimal alignment of instruction. The recent theoretical compilations and systematic evaluation of framework suggest that selection of simulation methods being driven by clearly defined instructional objectives is essential for the development of meaningful educational outcomes and the efficient application of resources. More specifically the various types of simulation should be systematically aligned with educational goals, which should be clearly operationalized through available frameworks (42). Further, careful consideration of resource limitations, financial resources, available faculty expertise, institutional facilities, technological infrastructure, and feasibility for maintenance become required to ensure implementations, which will be sustainable and scalable over time (116). With respect to fidelity of simulations however, it is suggested that functional alignment supersedes that of mere visual realism. There is ample evidence, which indicates that functional fidelity, by inference defined as the accurate reproduction of critical task features and cognitive mechanisms necessary for the acquisition of target accomplishments, leads to more prolonged and enhanced educational benefits than that derived simply from pictorially realistic equipment (117). Therefore, it is incumbent upon educational administrators and course designers alike, to adequately align the fidelity of the simulation tasks with clearly defined educational and evaluative objectives and not seek expensive, yet visually sophisticated simulation modalities unnecessarily. Secondly the instructional design process must by necessity include considerations of evaluation from the beginning. Reviews of current simulation evaluation processes indicate that much dependence is given to superficial learner satisfaction indices in many existing courses. What is recommended instead is that there should be comprehensive evaluation alignment, with an incorporation of validated performance indices, clearly measurable competency levels, and systematic rater training protocols, introduced into the planning of educational instruction from the beginning. Educational designs of simulation training which are based upon rigorous evaluation methods favor the availability of the opportunity for rigorous evaluation achieved at higher levels of the Kirkpatrick evaluation method and allow that simulated accomplishment reflects, as expected, authentic clinical performance. In conclusion, then, a four-dimensional alignment framework as proposed affords a global and evidence-based approach to decision-making. This comprehensive alignment technique formulates a clear consistency between educational objectives, fidelity of simulation, availability of resources, and evaluative intensity. Both the educational quality and its sustainability are greatly improved. This structured approach to instructional design meets with not only programmed consistency to taught curriculum and clinical rotations, but is a powerful evidence-generating approach for programmatic decisions surrounding learner evaluation and competency-based entrustment.

### 5.7 Equity, accessibility, and inclusive simulation design

Equity in simulation-based education extends beyond the availability of low-cost simulation alternatives. The following measurable dimensions must be systematically addressed to ensure fair and inclusive access to high-quality simulation learning opportunities:

- Geographic access: Simulation resources are disproportionately concentrated in urban academic medical centres. Mobile simulation units, telehealth-integrated simulation, and regional simulation networks represent strategies to extend access to rural and remote settings (118, 119). However, evidence on the effectiveness and sustainability of these outreach models remains limited.
- Digital infrastructure: The increasing adoption of VR/AR, screen-based simulation, and AI-assisted feedback creates dependence on reliable high-bandwidth internet, compatible hardware, and technical support infrastructure. These requirements raise equity concerns in LMICs and underserved regions. A “digital simulation readiness” assessment should precede investment in technology-dependent modalities.
- Access to internet and technology: The digital divide—differential access to reliable internet connectivity and up-to-date devices—represents a structural barrier to equitable participation in technology-enhanced simulation. Offline-capable simulation tools and low-bandwidth alternatives should be prioritized in digitally underserved contexts.
- Accessibility for learners with disabilities: Universal Design for Learning (UDL) principles should be applied to simulation design, including physical accessibility of simulation centres and equipment, and accommodations for learners with visual, auditory, motor, or learning disabilities during simulation participation and assessment. Current evidence on disability-inclusive simulation practices is extremely limited.
- Linguistic and cultural adaptation: The predominance of English-language simulation scenarios, assessment instruments, and debriefing protocols limits accessibility for non-English-speaking learners and may introduce cultural biases in performance assessment. Culturally validated scenario content, linguistically appropriate debriefing, and culturally competent SP portrayals are needed.
- Faculty capacity: Simulation-trained faculty are concentrated in high-resource institutions. Faculty development programs—including train-the-trainer models, massive open online courses (MOOCs), and peer-coaching networks—represent equity interventions that build local capacity in underserved settings (107, 108).
- Resource distribution: Institutional simulation budgets should be allocated using equity-informed frameworks that consider learner need, population health priorities, and existing resource disparities rather than historical allocation patterns or departmental bargaining power.

Addressing these dimensions requires moving beyond a narrow focus on “low-cost” alternatives toward a comprehensive equity framework that informs simulation program design, resource allocation, and governance at institutional, regional, and national levels.

As elaborated in Section 7.5.2, these equity dimensions are operationalized through six governance mechanisms aligned with the 2024 Global Consensus Statement on Simulation-Based Practice in Healthcare, which positions equity, diversity, and social accountability as fundamental components of simulation practice (120).

## 6 Integration of evidence on simulation effectiveness: from learning outcomes to patient and system-level improvements

### 6.1 Level 1: learner reaction and satisfaction

Throughout the past five years, systematic reviews and meta-analyses that have been published consistently reveal moderate-to-large effect sizes supporting simulation-based education vs. non-simulation or traditional instructional techniques with commensurate increases in learner satisfaction and engagement (76). It is important to note that satisfaction and self-reported engagement represent Kirkpatrick Level 1 outcomes—they reflect learner perceptions and should not be interpreted as evidence of learning or clinical transfer. These outcomes are typically measured through post-simulation surveys and are subject to social desirability bias and the halo effect of novelty. Positive learner reactions are a necessary but not sufficient condition for educational effectiveness.

### 6.2 Level 2: knowledge, skills, and attitudes

For modalities of digital simulation, VR has been found to improve theoretical acquisition of knowledge, development of procedural skills and retention and learner satisfaction; however, there has not been consistently supported evidence in the current database regarding critical thinking skills (77). Meta-analyses which have specifically targeted virtual simulations seeking to develop clinical reasoning suggest greater efficacy if the simulations target patient management,last longer than 30 min, utilize multiple variations of the scenarios and interactively provide immediate feedback following the learner's completion of the scenario (78). In similar fashion, newer meta-analyses targeting nursing education suggest significant development of clinical decision-making ability following virtual and digital simulations (79). However, several systematic reviews highlight the lack of data regarding long-term retention, especially regarding the lack of consistent data on follow-up beyond five months, and insufficient evidence regarding the patterns, thresholds, and timing regarding the retention of skills and performance. Explicit recommendations regarding the optimal instructional intensity and intelligent times of spacing and retention techniques are thus needed (80).

Recent evidence partially revises this picture for resuscitation skills. A 2025 systematic review of adult basic life support (BLS) training among healthcare providers found that feedback-integrated training—automated manikin feedback, video feedback, and simulation-based feedback—maintained resuscitation competency for up to 12 months, with sustained improvements in compression depth, ventilation quality, and overall resuscitation performance, whereas traditional instructor-led training declined significantly after six months (121)(post-search reference used for contextual discussion only). These findings are consistent with the spaced-learning and digital feedback evidence reviewed above (104, 105) and indicate that 12-month retention is achievable when feedback devices and structured booster schedules are embedded in the instructional design rather than reliance being placed on annual, one-off training events.

Outcomes reported in this section are primarily objectively measured (knowledge tests, skills checklist scores, OSCE ratings, TEAM scale assessments). Self-reported self-efficacy or confidence, while frequently reported in the primary literature, is explicitly noted as such when cited, and readers are cautioned that self-reported measures may not correlate strongly with objectively assessed competence.

### 6.3 Level 3: behavioural transfer to clinical practice

Concerning the transfer of learning from simulation to clinical performance, a recent scoping review in nursing education shows that, although there is strong evidence supporting the effectiveness of simulation for the acquisition of skills and strategies, the evaluation of this transfer usually relies on short-term or self-report measures. There are few data on the long-term transfer to performance in the work environment, and it will be necessary to use objective, longitudinal measures to be able to confirm this transfer more satisfactorily (81). In contrast, recent systematic reviews of *in situ* simulation (ISS) involving nurses in practice demonstrate that ISS results in an improvement and maintenance of clinical competencies in the cognitive, psychomotor, and affective domains in acute care settings (82). At the team and organizational levels, simulation-based training in clinical event feedback improves greatly participant performance in the leading of structured feedback, and their comfort, self-reported, in actual workplace situations, indicating a functional transfer in subsequent performance in clinical practice (83). In addition, systematic reviews of the safety of the ISS in emergency departments affirm that it is feasible overall and presents minimal risk when properly structured, and this structured implementation entails institutional protocols and risk management systems with a view to minimizing interruptions in functioning (84).

It is essential to distinguish between performance demonstrated within a simulated environment and independently verified behaviour change in clinical practice. The former represents Kirkpatrick Level 2 (simulated performance), while the latter constitutes Kirkpatrick Level 3 (behavioural transfer). In this section, only evidence of the latter type is reported. Where behavioural transfer was measured through self-report rather than direct observation, this limitation is explicitly noted.

### 6.4 Level 4a: organizational process and system outcomes

Emerging evidence increasingly links simulation-based training to measurable healthcare-system process outcomes. In obstetric care, systematic reviews and meta-analyses indicate that multidisciplinary obstetric emergency team simulations are associated with potential reductions in brachial plexus injury and demonstrate favorable trends for reducing the occurrence of low five-minute Apgar scores (<7), though statistical significance has not yet been universally attained. *in situ* multiprofessional simulations appear particularly advantageous (85). Further, *in situ* emergency cesarean-section simulations consistently demonstrate reductions in decision-to-delivery intervals (DDI) and improvements in one-minute Apgar scores, establishing preliminary links between simulation-enhanced procedural efficiency and improved early neonatal process indicators (86). Systematic reviews in critical care similarly suggest ISS serves as a valuable strategy within patient safety improvement initiatives (87). From an organizational-culture perspective, simulation-based patient safety training for operating-room nurses improves compliance behaviors, perceptions of patient safety culture, and educational satisfaction, providing preliminary support for simulation's potential in fostering systemic cultural improvements (88).

These findings, derived predominantly from obstetric emergency and critical-care simulation studies, should not be generalized to the entire field of simulation-based education. Evidence in other clinical domains remains limited. Furthermore, clinical process indicators (e.g., DDI, guideline adherence) must be distinguished from direct patient outcomes (e.g., mortality, morbidity), which are addressed separately in Section 6.5.

### 6.5 Level 4b: patient outcomes

A small number of studies have investigated associations between simulation-based training and direct patient outcomes. In obstetric care, preliminary evidence suggests associations between multidisciplinary team simulation and reduced rates of brachial plexus injury and favorable trends in five-minute Apgar scores, although these findings did not consistently reach statistical significance and should be interpreted as hypothesis-generating rather than confirmatory (85). In critical care, a single-site pilot study of SBML for ventricular assist device self-care reported a decreasing trend in driveline exit site infections (5); however, this finding derives from a small, uncontrolled pilot study and should not be generalized. In infection prevention and control, systematic review evidence indicates that simulation-based education is associated with improved skill acquisition and attitude formation among healthcare students, while knowledge retention was comparable to traditional didactic methods (114).

Overall, evidence directly linking simulation-based education to definitive patient outcomes (mortality, major morbidity, hospital-acquired complications) remains methodologically limited. The majority of evidence is derived from selected clinical areas—primarily obstetrics and critical care—and is characterized by small sample sizes, single-site designs, lack of blinding, and short follow-up periods. The inference that simulation improves patient outcomes, while plausible and supported by mechanistic reasoning (improved skills → improved clinical performance → improved outcomes), should be advanced cautiously and with explicit acknowledgement of the limitations of the current evidence base. Statistically non-significant trends (e.g., in Apgar score distributions) have been described as such throughout this section and are not presented as evidence of benefit. Perceptions of patient safety culture are reported separately from direct patient outcome data, acknowledging that safety culture perceptions represent a distinct construct measured through self-report instruments (88).

### 6.6 Influencing factors, moderators, and mediators

Debriefing quality continues to emerge as one of the most influential moderators impacting simulation effectiveness. Recent methodological syntheses and reviews emphasize that structured debriefing processes, facilitator communication strategies, and psychological safety management significantly correlate with learning outcomes, though empirical evidence quality varies, and results remain highly context-sensitive. This underscores the critical need for consistent debriefing frameworks and systematic faculty development programs (90). Regarding participant role assignments within simulations, emerging evidence suggests observer roles are not inherently ineffective; however, active learner participation tends to yield superior outcomes in terms of self-efficacy and retention of procedural skills, with the magnitude of these differences influenced by task complexity and time intervals between training and assessment (90). Regarding optimal instructional dosage and pacing, accumulating evidence consistently supports deliberate practice schedules involving iterative practice–feedback–repractice cycles with predefined mastery standards. Simulation duration and structured debriefings exhibit synergistic interactions enhancing educational effectiveness. Importantly, evidence suggests prioritizing functional and psychological fidelity over superficial visual realism for maximizing learner outcomes (91). Furthermore, learner baseline abilities, multiprofessional team composition, and scenario complexity interactively influence cognitive load, thus recommending integrated instructional designs that carefully balance task complexity, cognitive load, and structured debriefings rather than isolated optimizations (75).

### 6.7 Methodological heterogeneity and research quality

Methodological heterogeneity remains a critical limitation restricting evidence synthesis and interpretability. The Medical Education Research Study Quality Instrument (MERSQI) has recently undergone proposals for revision to improve the inclusion and weighting of essential research design elements, thereby fostering enhanced comparability and reproducibility of educational research findings (75). Within recent meta-analyses, publication-bias assessments and sensitivity analyses generally appear acceptable, although variability in study quality, limited follow-up durations, and unstable effect sizes constrain external validity and generalizability of conclusions. Some RCT meta-analyses have reported negligible publication biases and relatively robust sensitivity results (76). Methodologically, future meta-analytic reviews should incorporate explicit robustness tests, including sensitivity analyses examining the impact of unpublished negative or null-effect studies and publication bias considerations, thereby increasing the robustness of conclusions under varying analytical assumptions (122). For non-randomized educational interventions, the systematic application of Risk of Bias In Non-Randomized Studies of Interventions (ROBINS-I) tools is advised to clarify potential bias pathways and evidence-certainty implications.

### 6.8 Critical evidence gaps and areas of uncertainty

While significant contributions have been made in the interim, important lacunae exist in the present evidence base, limiting a full understanding of the efficacy of simulation-based education. Long-term retention and skill transfer remain unknowns, and there is a dearth of follow-up data extending longer than five or six months postintervention. Recent feedback-integrated BLS training studies, however, demonstrate 12-month maintenance of resuscitation competence (121), indicating that prolonged retention is achievable for specific skills under structured feedback and booster conditions; comparable long-term data remain scarce for most other technical and non-technical skill domains. Further, empirical descriptions of curves of skill retention including rates and decay patterns are few, indicating future longitudinal study (80). With respect to patient-centered outcomes, while certain clinical areas, e.g., obstetrics, have received preliminary data of a suggestive nature, the overall rigor of the evidence base in professional medical disciplines is poor,concluding for the necessity for proper multicenter investigations and standardized real-world evaluations with useful parametric measures of patient outcomes (123). Barely several studies systematically fashion any economic study component into their investigations, thus challenges have been launched for the systematic elaboration of cost-effectiveness variables and dollars-and-cents projections into early stage study design with regard to arriving at optimal numbers for training duration and frequency as a product of economic analysis and break-even study (19). Recent exceptions are emerging: the health-system ROI analysis of rapid ICU workforce upskilling (73) and structured cost-effectiveness evaluations in primary care procedural skills (74) demonstrate that rigorous economic evaluation is feasible when designed prospectively, as elaborated in Section 7.4. Issues of equity and access are another dimension of great importance but which are often ignored. A global consensus statement underscores the urgency for simpler to learn, relatively inexpensive, replicable simulation methods and faculty development programs which will be available broadly. An environment is called for which will have a thorough governing framework focusing upon issues of safety, security, fairness in access to financial and intellectual resources, and distributive equity in the availability of resource supply (120). Further, clarification is needed as to whether strategies regarding instruction and educational objectives are congruent. Thus, one looks at review literature with respect to infection control education and finds that simulation methods may be superior to acquisition of skills and formation of attitudes in educational objectives, while knowledge retention is aided by the traditional didactic methods of instruction, indicating the necessity of understanding how to align instruction with objective (114). The greater uptake and sustaining of emergent technologies, e.g., VR/XR simulations will be in large areas dependent upon hospital and organizational/regulatory culture, checking attitudes of acceptable performance, attainability and usability of technology, and structures of settlement with regard to aid deficit supply, all indicating the pressing need for further empirical investigation and studies which are directed toward implementation (124). And, finally, in those areas of provision for medical education where resources are limited, it is to be anticipated that the development and progressive elaboration and validation systems of competence as they refer to accreditation study of simulation instructors will be numbered among the important missions of the educational interests of simulation-based educational programs, insuring the possibility for educational achievement, and the progressive implementation of simulation-based training in resource-scarce environments (125). Recent 2024–2025 evidence is beginning to narrow these gaps: a health-system economic analysis reported a 478% return on investment for ICU workforce upskilling (73), and feedback-integrated training has demonstrated 12-month resuscitation-skill retention (121). The critical gaps in evidence which have been identified must be closed by careful and integrated and directed investigation in the future to promote substantially both the theoretical within the instance described and the practical accomplishment of simulation-based educational studies.

## 7 Implementation and translational practice

### 7.1 Conceptual boundaries: differentiating translational simulation subtypes

Before discussing implementation science frameworks, it is necessary to distinguish among related but conceptually distinct approaches to simulation that operate at different levels of the healthcare system:

- Educational simulation: Simulation whose primary purpose is individual or team competency development—i.e., teaching and assessing clinical knowledge, skills, and attitudes. The majority of published SBE falls within this category.
- Simulation-based clinical systems testing (SbCST): The deliberate, prospective use of simulation to evaluate clinical environments, workflows, equipment, and processes before they are used with real patients. SbCST primarily identifies latent safety threats (LSTs)—system vulnerabilities that could compromise patient safety but have not yet caused harm (126, 127).
- Identification of latent safety threats (LSTs): A specific output of SbCST and translational simulation. LSTs are system-level hazards (e.g., missing equipment, ambiguous protocols, incompatible workflows) detected through systematic simulation-based probing of clinical environments and processes.
- Simulation for quality improvement (SimQI): The integration of simulation into formal quality improvement methodologies (e.g., Plan-Do-Study-Act cycles, Lean, Six Sigma) to iteratively test and refine clinical processes. SimQI explicitly links simulation activities to measurable process improvement outcomes (128).
- Translational simulation: A broader conceptual model (Brazil & Reedy, 2024) in which simulation is used diagnostically—as a probe to investigate clinical systems, team performance, and organizational culture—directly linking educational activities to patient and system-level quality and safety outcomes (67). Translational simulation encompasses SbCST, LST identification, and SimQI within a unified framework.

These distinctions are maintained throughout Section 7 to prevent conflation of educational and systems-testing functions of simulation.

### 7.2 Application of implementation science frameworks

Transitioning simulation-based education from isolated teaching activities to comprehensive organizational integration primarily requires contextual adaptation informed by established implementation frameworks. The updated Consolidated Framework for Implementation Research (CFIR) underscores systematically identifying implementation barriers and facilitators across five critical domains—intervention characteristics, external environment, internal organizational context, individual participant factors, and process dynamics—while emphasizing equity considerations and stakeholder perspectives. Recent CFIR implementation guidelines outline structured five-step protocols accompanied by practical tools, enabling explicit alignment of simulation initiatives with identified implementation factors and appropriate strategies (19, 65). Complementarily, the RE-AIM and PRISM frameworks prioritize comprehensive assessments encompassing participant reach, intervention efficacy, organizational adoption, fidelity of implementation, and sustained maintenance, collectively addressing scalability and sustainability. Empirical studies and reviews published in 2025 illustrate that structured RE-AIM applications effectively quantify population engagement, organizational uptake, implementation fidelity, and sustained adoption, significantly enhancing external validity and replicability—thus making these frameworks particularly suitable for systematic evaluation and dissemination of hospital-based or regional simulation projects (20, 66). Translational Simulation further integrates educational outcomes with systems-level quality improvements, utilizing simulations proactively as systemic probes to validate process enhancements, thereby effectively informing subsequent RE-AIM evaluations (67).

Table 2 presents a structured CFIR analysis of five illustrative implementation cases from the reviewed literature, mapping identified barriers and facilitators across the five CFIR 2.0 domains. For example: (a) Intervention characteristics—the complexity of interprofessional hybrid simulations was identified as a barrier to adoption; modular, protocolized simulation packages with scripted facilitator guides served as facilitators (18, 60). (b) Inner setting—organizational leadership support and dedicated simulation coordinator positions emerged as critical facilitators (129, 130). (c) Outer setting—accreditation requirements and regulatory expectations (e.g., ACGME milestones, CanMEDS EPAs) drove adoption but also raised concerns about “teaching to the test” (130, 131). (d) Individual characteristics—faculty self-efficacy with debriefing facilitation and learner psychological safety were identified as key individual-level determinants (31, 107). (e) Process—iterative Plan-Do-Study-Act cycles embedded within translational simulation programs facilitated sustained implementation (67, 128). (See Table 2 and Supplementary Table S3 for detailed RE-AIM/PRISM evaluation matrices across reach, effectiveness, adoption, implementation fidelity, maintenance, cost, and scalability dimensions.)

Table 2. Structured CFIR 2.0 analysis of illustrative simulation-based education implementation cases from the included literature. The table maps key barriers and facilitators to implementation across the five core CFIR 2.0 domains, with illustrative case examples anchored to empirical findings from the review's included studies. This analysis moves beyond descriptive presentation of the CFIR framework to apply the model analytically to real-world simulation implementation scenarios, in response to reviewer feedback.

### 7.3 Curriculum integration and pathways to scalability

Effective scaling of simulation-based education involves more than increasing simulation frequency; rather, simulations must become deeply embedded within Competency-Based Medical Education (CBME) and comprehensive programmatic assessments. Institutional-level implementations, such as major facility relocations, increasingly utilize translational simulation strategically within formal change management frameworks, underscoring simulation's utility in aligning systemic workflows and processes (129). In undergraduate medical education, repeated cycles employing Objective Structured Clinical Examinations (OSCEs) combined with simulation-based evaluations of communication and clinical reasoning competencies have generated longitudinal transitional evidence bridging the gap from competency demonstration to clinical performance (132). In postgraduate contexts, national surveys among Canadian emergency medicine residency programs indicate that CBME frameworks effectively drive the integration of simulation experiences with clearly defined EPA assessments, while concurrently highlighting the necessity of balancing rigorous assessment with psychological safety considerations (130). Additionally, the University of Washington's programmatic assessment reforms exemplify explicit incorporation of longitudinal evidence collection, triangulation methods, and proportionality principles, systematically linking simulation-derived evidence to committee-level entrustment decisions (133). National surveys across U.S. undergraduate medical programs further confirm widespread adoption of simulation-based education, yet identify substantial opportunities for expanding simulation's role in developing advanced communication, empathy, and higher-order reasoning skills (131). Therefore, curriculum designers are advised to systematically construct evidence-informed progression frameworks explicitly linking defined competency milestones to corresponding assessmentevidence, ensuring alignment of instructional strategies with educational objectives and proactively mitigating unintended learning consequences associated with high-stakes assessments. Curriculum integration should therefore be conceived as a design problem rather than a scheduling problem. In Backward Design terms, simulation units are planned backward from EPA-anchored outcomes to scenario design (41); in ADDIE terms, each cohort iteration feeds the Evaluate phase of the next cycle (42) (Section 3.6.1). This alignment connects simulation curricula to the programmatic assessment principles described above and prevents the accumulation of simulation hours without corresponding competency progression.

### 7.4 Quality assurance and continuous improvement

The integration of simulation into organizational quality assurance necessitates explicit alignment with Learning Health Systems (LHS), as well as adherence to paradigms of continuous quality improvement. A structured five-step quality improvement framework has recently been proposed which explicitly applies simulation methods to systematically address critical clinical incidents, embedding formal quality improvement methods within translational simulation programs to improve post-event response, engender organizational learning, and promote a sustained trajectory of improvement (128). In addition, applied clinical simulation testing (SbCST) simulation prior to large facility openings or physical reallocation, in concert with this, provides an effective means of identifying latent safety threats (LSTs), as well as optimizing team readiness, permitting prevention and proactive management of risk (126). Human factors simulation has likewise facilitated a holistic evaluation of Electronic Health Record (EHR) system utilization and optimization, in a manner that identifies workflow vulnerabilities through realistic scenario simulations, thus directly informing pre-implementation refinements of the process (134). Recent complexity-based frameworks advocates the explicit positioning of simulation as instruments of systemic change, promoting and enhancing consistency of organizational learning, interdisciplinary collaboration, context-specific variability and iterative process refinement (135). These frameworks again provide congruence with present LHS methodologies, including the use of bona fide quality indicators in the context of artificial intelligence, specifically with a view to the iterative cycles of data-driven organizational improvement and strategic governance alignment (68). Accordingly, organizations are correctly advised to establish comprehensive dual-tiered metrics encompassing process orientated variables, such as reach of participants, fidelity of implementation, quality of debriefs, LSTs identified and resolution rates, as well as outcome oriented measures, such as skill mastery, skill retention, clinical process efficiency, clinical transfer variables, and safety event tracking, as well as continued dissemination of the performance outcomes, processes and improvement modalities identified. Two further elements should be incorporated into this dual-tier metric system. First, latent safety threat (LST) identification and resolution rates should be tracked as leading indicators of system learning, alongside debrief quality (126, 127). Second, the metrics should be equity-disaggregated—process and outcome indicators reported by learner group—to detect differential benefit across populations, consistent with the EDI governance mechanisms proposed in Section 7.5.2 (71, 120).

### 7.5 Economic evaluation and cost-effectiveness considerations

Economic evaluations of simulation programs should provide an extensive examination of the entire educational value chain, extending past basic acquisition costs to explicit measurement of training efficiency, translation of skills to practice, and associated healthcare outcomes. Five categories of economic evaluation must be clearly distinguished:

- Cost description (CD): Systematic enumeration of all direct and indirect costs associated with a simulation program, without comparative analysis against alternatives.
- Cost-effectiveness analysis (CEA): Comparison of costs to a single natural-unit outcome (e.g., cost per additional competent learner, cost per procedural error avoided, cost per life-year gained).
- Cost-utility analysis (CUA): Comparison of costs to preference-weighted outcomes [e.g., cost per quality-adjusted life year [QALY] or disability-adjusted life year [DALY]].
- Return-on-investment analysis (ROI): Calculation of net financial return (benefits minus costs, divided by costs), expressed as a ratio or percentage.
- Budget-impact analysis (BIA): Projection of the total financial impact of adopting a simulation program on a specific institutional or health-system budget over a defined time horizon, accounting for both implementation and ongoing operational costs.

Single-study positive findings from any of these five economic-evaluation types cannot be generalised to demonstrate broad economic viability of simulation-based education. Estimates are highly context-dependent, and sensitive to local staffing expenses, equipment procurement costs, trainee volume, overhead expenditure, and organisational implementation capacity. Extrapolation across different settings should be made with great caution.

A recently advanced value-based simulation framework in health care directly aligns simulation-derived outcomes with defined objectives, requiring detailed clusters of actionable indicators regarding efficiency, quality of care, patient safety, productivity of the health care workforce, and financial outcomes, which will guide initial investment decisions and subsequent outcome evaluations (21). The analytical framework of this review accommodates the shift toward Value-Based Simulation in Healthcare (VBSH) in three specific ways. First, value is treated as a distinct analytic layer rather than a terminal Kirkpatrick level: Sections 6.4 and 6.5 map simulation onto organizational process and patient outcomes, while the present section maps the same activities onto financial and value metrics, so that value is assessed across, not instead of, the Kirkpatrick-Phillips hierarchy. Second, the VBSH indicator clusters—efficiency, quality of care, patient safety, workforce productivity, and financial outcomes (21)—map directly onto the dual-tier metrics proposed in Section 7.3 (process tier) and the outcome measures of Sections 6.1–6.5 (effectiveness tier), yielding the operational dashboard through which value, rather than activity volume, is monitored. Third, the selection matrix (Table 1) embeds value considerations at the point of instructional design by linking each clinical task to functional-fidelity, low-cost alternatives wherever the evidence supports equivalence (Section 5.5), making cost-conscious design the default rather than an afterthought. VBSH is therefore conceptualized here not as a replacement for Kirkpatrick-based evaluation, but as a value layer that builds on Levels 3–4b evidence while supplying the financial and workforce-productivity indicators that funders and health systems require. Systematic reviews of economic evaluations in undergraduate medical education have documented persistent variability in regard to the definitions of cost boundaries and transparency of methodology, recommending explicit inclusion of sensitivity analyses and total budget-impact analysis to enhance the rigor of economic evaluations (72). A single health-system-level economic modeling of rapidly developed skill acquisition programs for ICUs, applied at the healthcare-system level, has demonstrated ample return on investment (ROI), suggesting potential economic viability under the specific conditions studied (73). Specifically, this economic analysis of a rapid ICU workforce upskilling program—17,494 non-ICU professionals trained across 24 countries—reported a deterministic return on investment of 478%, with programme costs fully recovered within 5.1 days; probabilistic sensitivity analysis (10,000 Monte Carlo iterations) confirmed robustness, with a mean ROI of 455% (95% CI 130–1,029%) (73). However, these findings should not be generalized as evidence of broad economic viability across all simulation programs and settings. Similarly, instruction in procedural skills in primary care likewise documents favorable cost-effectiveness results, associated with measurable increases in learner skills and self-efficacy (74). Economic evaluations, therefore, should specifically include comprehensive cost inventories, systematic examination of clinical translatability efficiencies, ROI analyses, total cost-effectiveness/cost-utility analyses, sensitivity analyses, and ongoing, annual budget-impact analyses. Positive findings from individual economic evaluations must be contextualized within their specific settings and methodologies rather than generalized. Consistent with VBSH, prospective economic evaluations should embed value-based indicator clusters from programme inception, alongside total cost, sensitivity, and budget-impact analyses (21, 72).

### 7.6 Governance and ethical considerations

In the governance frameworks developed for simulation programs, there is a need to strongly emphasize safety, compliance with regulations, equity, and transparency. Systematic reviews on ethics in simulation emphasize that there needs to be an assurance that learner well-being and psychological safety are considered throughout the simulation process and prevent exposure to embarrassment or psychological harm, and require strict adherence to ethical practice in reporting outcomes (22, 136). This is also the time that Equity, Diversity, and Inclusion (EDI) frameworks now exist and promote the development of competencies enabling educators to more consciously include awareness of social justice, and their impact on cultural diversity and inclusive opportunities within the design, implementation and reflective debriefing in simulation (71). In the area of data governance, contemporary models of ethical frameworks in the AI environments, promote “co-created informed consent” models, which enable balancing the privacy protection of individuals with the needs of the legitimate use of data, as well as suggesting specific legal and ethical frameworks to encourage anonymization and safe practices of collecting, managing and storing, recorded audiovisual, text, and log data (69, 70). Systematic reviews also exist that synthesize up-to-date psychological safety practices and language protocols in advanced practice populations, producing ready-built educational modules and organizational strategies that are transferable across professional groups (32). It is therefore vital for educational bodies to develop written, specific standard operating procedures (SOPs) that detail protocols on ethics of consent and confidentiality, audiovisual recording, secure data storage, transparency in appeal methods for AI-provided feedback, debriefing norms, and reporting of psychological safety and equity metrics, as valid determinants of good governance.

#### 7.6.1 The partnership principle: application to AI governance and feature bloat

To operationalize these governance requirements, we propose a partnership principle as the organizing logic of simulation governance: governance structures should be co-designed and co-owned by the stakeholders who create, deliver, and experience simulation—learners, educators and facilitators, clinical service leaders, institutional quality and safety officers, and, where feasible, patient and public representatives (65, 67, 120).

Applied to AI ethics, the partnership principle mitigates the risks identified in Section 4.7.1 through four concrete mechanisms: (a) learners and educators jointly define acceptability thresholds for AI-generated feedback through co-created informed consent models for data use (69); (b) appeal mechanisms are designed with learner representation, so that AI-generated assessments can be challenged and receive human review without fear of reprisal; (c) routine bias audits of AI assessment outputs are reviewed by a governance group that includes end-users, ensuring that disparate impact is detected by those affected rather than by developers alone; and (d) professional trust is treated as a governance asset, with AI outputs presented as provisional and verification obligations assigned to named humans—a direct response to the warning that bias, hallucination, and emergent unpredictability can otherwise corrode trust in simulation systems (115).

Applied to technology adoption, the partnership principle operates as a structural check on feature bloat: no new simulation technology—AI-enabled or otherwise—is procured or deployed unless it can be mapped to at least one defined learning objective and one measurable outcome indicator within an instructional causal chain (Section 3.6), with staged evaluation against value-based metrics (21) preceding organization-wide rollout. This procurement discipline directly addresses the recurring failure mode in which visually sophisticated or AI-branded equipment is adopted as a proxy for instructional quality (Section 4.2).

#### 7.6.2 Equity, diversity, inclusion, and social accountability in institutional governance

The 2024 Global Consensus Statement on Simulation-Based Practice in Healthcare positions equity, diversity, and social accountability as fundamental components of simulation practice rather than peripheral environmental concerns (120). Accordingly, we propose six specific mechanisms through which institutions can embed the Consensus Statement in simulation governance (Table 3): (1) a standing EDI item on the simulation governance committee agenda, with defined metrics for participation, scenario representation, and differential outcomes across learner groups; (2) periodic EDI audits of scenario banks, assessment instruments, and debriefing language protocols to detect stereotyped portrayals, culturally biased scoring, and linguistically inaccessible content (32, 71, 120); (3) a social accountability charter aligning a defined proportion of simulation activity with community health priorities and underserved settings, including low-cost and tele-simulation outreach models (118, 119); (4) learner and community representation in governance, so that those affected by inequity participate in decisions rather than being consulted after the fact; (5) equity-weighted resource allocation replacing historical budgeting patterns with needs-based criteria (Section 5.7); and (6) mandatory faculty development in EDI-informed debriefing, enabling facilitators to address equity-relevant performance events without stigma (32, 71). These mechanisms convert the Consensus Statement's principles into auditable governance practices.

### 7.7 Illustrative translational cases and lessons learned

Specific translational implementations offer key insights for future simulation practice. In large institutional transformations, translational simulation has proven critical in structured change management strategies, permitting complete systematic assessments, workflow optimization, and improved staff readiness through proactive governance and effective organizational communication (137). Prior to the opening of pediatric intensive care units, structured SbCST effectively surfaced and mitigated latent threats to safety, permitting correction prior to the commencement of clinical operations (127). In resource-constrained rural settings, telehealth-integrated simulation initiatives supported structured capacity building for community instructors to conduct *in situ* role-play scenarios, permitting significant improvements in local preparedness and resource utilization for critical maternal emergencies (118, 119). Simulation-based pre-implementation testing of clinical processes and early warning systems has similarly surfaced critical workflow and human factors vulnerabilities, permitting pre-emptive process optimization and optimal clinical transition (138). Key success factors for these translational initiatives are consistently clearly articulated problem-oriented goals, interdisciplinary collaboration among appropriate stakeholders, rigorous structured debriefings resulting in iterative cycles of improvement, and comprehensive multidimensional RE-AIM evaluations. A detailed RE-AIM/PRISM evaluation matrix for each illustrative case is provided in Supplementary Table S3.

#### 7.7.1 Clinical practice illustrations: team dynamics, role allocation, and error management in time-critical settings

To make the theoretical framework directly usable by clinicians, we present three worked illustrations—anchored to the included literature—of how team dynamics, role allocation, and error management operate in time-critical acute care and pediatric emergency settings, and how structured debriefing addresses each.

Case 1—Pediatric resuscitation: time-critical teamwork under high stakes. In pediatric emergency team training, systematic review evidence shows that simulation improves adherence to time-critical actions and clinical guidelines, including resuscitation algorithms, although effects on definitive outcomes such as survival remain of limited certainty (4). Role allocation is the dominant design variable: crisis resource management (CRM)-based programs that assign explicit resuscitation roles (team leader, airway, compressions, medication, scribe/timer) improve participants' understanding of role assignments and their perceived value of structured debriefing (58). Error management in this setting targets both technical errors (compression quality, drug dosing) and coordination errors (role confusion, open-loop communication). Structured debriefing addresses these through the PEARLS phases: the reaction phase normalizes emotional responses; the description phase reconstructs the event timeline, revealing where role boundaries dissolved; the analysis phase uses advocacy-inquiry to examine why closed-loop communication failed at specific moments; and the summary phase consolidates take-home messages (30, 98). Evidence from clinical event debriefing further shows that simulation-based training improves leadership performance in pediatric emergency care, supporting the transfer of debriefing skills to real resuscitation events (83).

Case 2—Emergency cesarean delivery: multidisciplinary coordination under time pressure. In obstetric emergencies, *in situ* multiprofessional simulation consistently reduces decision-to-delivery intervals and improves one-minute Apgar scores, establishing preliminary links between simulation-enhanced procedural efficiency and early neonatal process indicators (85, 86). The team-dynamics challenge is coordination across disciplines with different habitual communication styles (obstetrics, anesthesia, midwifery, neonatology); the role-allocation challenge is clarity of the “who calls, who leads, who documents” boundaries during the decision-to-delivery window. Error management emphasizes latent system failures over individual blame: simulation-based clinical systems testing (SbCST), as used before pediatric ICU openings, surfaces missing equipment, ambiguous protocols, and incompatible workflows before patients are exposed (126, 127). Debriefing after each drill converts identified coordination failures into protocol revisions, closing the loop between simulation and system improvement (128).

Case 3—Crisis procedures in critical care and emergency medicine: mastery under pressure. For time-critical procedural skills (emergency cricothyroidotomy, chest tube insertion, emergency thoracotomy), SBML with defined MPS and coached re-practice achieves universal skill acquisition and reduces performance variability (27, 45), while low-cost task trainers make such training feasible in resource-limited settings (63, 64). Role allocation extends to observers: emerging evidence suggests observer roles are not inherently ineffective, but active participation yields superior self-efficacy and skill retention, with the magnitude influenced by task complexity and training–assessment intervals—a design consideration for rotation logistics (90). Error management is engineered into the design: zero critical errors constitutes the MPS threshold, and spaced retention checks detect skill decay before clinical exposure. Structured debriefing (TeamGAINS, advocacy-inquiry) follows each practice cycle to connect performance gaps to decision-making rationale rather than motor execution alone (57).

Across these cases, structured debriefing functions as the common remediation mechanism for all three problem classes: team dynamics are addressed by analyzing communication patterns against explicit models; role allocation is addressed by comparing enacted roles against the predefined role map; and error management is addressed by reconceptualizing errors as system-level learning opportunities within a psychologically safe frame (30, 31). The PEARLS framework, standardized through faculty development, ensures this remediation is delivered consistently rather than varying with individual facilitator skill (98).

### 7.8 Synthesis: implementation gaps and scalability challenges

Cross-cutting analysis of the implementation literature reveals several persistent gaps: (a) the majority of implementation research in SBE has been conducted in high-resource academic centres, limiting generalizability to LMIC and community settings; (b) implementation fidelity is rarely systematically measured or reported; (c) the sustainability of simulation programs beyond initial grant-funded or pilot periods is poorly documented; (d) the cost data necessary for budget-impact analyses are frequently absent; and (e) the translation from simulation-based competency demonstration to sustained clinical behaviour change and measurable patient benefit remains inadequately evidenced. Addressing these gaps will require prospective, multi-site implementation studies with embedded economic evaluation and longitudinal clinical outcome assessment, guided by CFIR, RE-AIM/PRISM, and Translational Simulation frameworks.

## 8 Conclusion

The efficacy of simulation-based education is conditional on the alignment of educational aims, instructional design, assessment methodology, and implementation context. Moderate-to-high-certainty evidence demonstrates that SBE is associated with improvements in knowledge acquisition, procedural skills, clinical reasoning, and team-based non-technical skills when the following conditions are satisfied: alignment between learning objectives, activities, and assessment; integration of structured, high-quality debriefing sessions; mastery-based instructional pacing; and the provision of psychologically safe learning environments. The evidence consistently indicates that functional and psychological fidelity should be prioritized over visual or technological sophistication—learning gains depend more on task-relevant design and learner engagement than on equipment complexity.

Translational simulation frameworks, analysed through CFIR and RE-AIM/PRISM, offer a systematic approach to linking educational interventions with clinical workflow improvements and organizational quality outcomes. Preliminary evidence from selected clinical areas (predominantly obstetrics and critical care) suggests associations between simulation-based training and improvements in clinical process indicators such as decision-to-delivery intervals, guideline adherence, and latent safety threat identification. Evidence for direct patient outcomes (mortality, major morbidity) remains methodologically limited—characterized by small sample sizes, single-site designs, and short follow-up—and should not be generalized across the entire field of simulation-based education. Statistically non-significant trends have been reported as such and are not interpreted as evidence of benefit. Economic evaluation evidence is similarly preliminary; positive ROI findings from individual studies should not be interpreted as evidence of broad economic viability. Recent analyses nonetheless demonstrate that favourable returns are achievable under defined conditions: the 478% ROI reported for rapid ICU workforce upskilling (73) and feedback-integrated resuscitation training sustaining competence at 12 months (121) establish empirical anchors for value-based evaluation.

The principal limitations of the evidence base include: (a) a paucity of long-term retention data beyond five to six months post-intervention, although feedback-integrated designs now demonstrate 12-month maintenance for resuscitation skills (121); (b) limited empirical data on skill decay curves and optimal refresher timing; (c) over-reliance on self-reported outcome measures at the expense of independently observed clinical behaviour; (d) concentration of evidence in high-resource academic settings, with limited generalizability to LMIC, rural, and community contexts; (e) insufficient integration of economic evaluation and equity metrics into SBE research; and (f) a near-absence of validated AI governance frameworks for simulation-based assessment.

Implications for practice: Educators should anchor simulation design in explicit competency frameworks (EPAs, CBME, QSEN) with structured pre-briefing and debriefing as central, non-negotiable components. Assessment should employ validated, direct observational instruments (behaviourally anchored ratings, temporal metrics, learning analytics) rather than relying predominantly on learner satisfaction surveys. Instructional intensity should be individualized to observed learning curves, with spaced reinforcement schedules informed by retention data. Administrators should adopt CFIR, RE-AIM/PRISM, and Translational Simulation frameworks to guide systematic implementation, evaluate scalability, and embed simulation within institutional quality improvement and Learning Health System cycles. Comprehensive economic evaluations—distinguishing among CD, CEA, CUA, ROI, and BIA—should be integrated prospectively into simulation program design. AI-assisted feedback and automated assessment should be treated as emerging, investigational tools reserved for formative feedback contexts and subject to rigorous human oversight, bias auditing, and transparent appeal mechanisms. Equity must be addressed multidimensionally—encompassing geographic access, digital infrastructure, disability accessibility, linguistic and cultural adaptation, faculty capacity building, and equitable resource distribution—rather than being reduced to the availability of low-cost alternatives.

The potential of simulation-based education to contribute to educational quality improvement and, where supported by rigorous evidence, to selected patient and system-level outcomes, depends on sustained commitment to instructional alignment, debriefing excellence, evaluative rigor, human-AI collaborative models, and robust empirical grounding in cost-effectiveness and equity-oriented research. These represent the foundations for scalable, sustainable, and ethically governed SBE that bridges the gap from educational activity to meaningful clinical and organizational benefit.
